# Footwear microclimate and its effects on the microbial community of the plantar skin

**DOI:** 10.1038/s41598-021-99865-x

**Published:** 2021-10-13

**Authors:** Te Miao, Peihua Wang, Nan Zhang, Yuguo Li

**Affiliations:** 1 Department of Mechanical Engineering, The University of Hong Kong, Pokfulam Road, Hong Kong, SAR China; 2 School of Public Health, The University of Hong Kong, Pokfulam, Hong Kong, China; 3 College of Architecture and Civil Engineering, Beijing University of Technology, Beijing, China

**Keywords:** Quality of life, Mechanical engineering, Bacterial development

## Abstract

The association between the footwear microclimate and microbial community on the foot plantar skin was investigated by experiments with three participants. Novel methods were developed for measuring in-shoe temperature and humidity at five footwear regions, as well as the overall ventilation rate inside the footwear. Three types of footwear were tested including casual shoes, running shoes, and perforated shoes for pairwise comparison of footwear microclimate and corresponding microbial community on the skin. The major findings are as follows: (1) footwear types make a significant difference to in-shoe temperature at the instep region with the casual shoes sustaining the warmest of all types; (2) significant differences were observed in local internal absolute humidity between footwear types, with the casual shoes sustaining the highest level of humidity at most regions; (3) the perforated shoes provided the highest ventilation rate, followed by running and casual shoes, and the faster the gait, the larger the discrepancy in ventilation rate between footwear types; (4) the casual shoes seemed to provide the most favorable internal environment for bacterial growth at the distal plantar skin; and (5) the bacterial growth at the distal plantar skin showed a positive linear correlation with the in-shoe temperature and absolute humidity, and a negative linear correlation with the ventilation rate. The ventilation rate seemed to be a more reliable indicator of the bacterial growth. Above all, we can conclude that footwear microclimate varies in footwear types, which makes contributions to the bacterial growth on the foot plantar skin.

## Introduction

Footwear is an irreplaceable part of modern human life given the necessity to protect our feet. However, while taking advantage of this function, the wearer is also subject to thermal discomfort and hygienic problems due to the enveloping of the foot. Similar to clothing, footwear prevents heat and water vapor from transferring freely to the external air, creating an enclosed environment with a high temperature and humidity^1^. Such a microclimate causes the shod foot to maintain a higher skin temperature and humidity than a barefoot due to decreased convective heat/mass transfer and sweat dissipation^2,3^. A relative humidity of 96–100% inside footwear may substantially contribute to bacterial growth and colonization of yeast-like fungi^4^. The former can stimulate common bacterial infections like erythrasma^5^ and contribute to foot odor due to degradation of the leucine present in sweat by *Staphylococcus epidermidis*^6^, while the latter can lead to fungal infections like dermatophytosis^7^. Most of the microbes and pathogens that are normally present in or on the human body are mesophiles, and grow best at around 37 °C^8,9^, which happens to be the peak value of the measured in-shoe temperature (27–37 °C) under constant moderate climatic conditions^10^. A decrease in the in-shoe temperature should inhibit the growth of microbes. Therefore, both in-shoe temperature and humidity should have a positive impact on the microbial populations of the foot skin, which would result in various hygienic problems. However, there is no adequate scientific evidence to support this association, although some qualitative results were obtained in an earlier study^11^.

For measuring the footwear microclimate, some researchers used sensors inside footwear and attached them on the foot surface at different locations, such as the toe, metatarsal, arch, heel, and the instep^10,12–14^. Others installed instrumented insoles with several integrated sensors on the insole surface^15–17^. These existing methods suffer from measurement inaccuracy as the motion of the foot inside footwear can be disturbed due to the reduced space, and the direct contact between the sensors and foot implies that the measurement might be partially influenced by the foot surface rather than the air environment.

An important aspect of the footwear microclimate is the internal ventilation rate, which also provides an indicator for the convective heat/mass transfer inside the footwear. The air exchange between the footwear environment and the outside environment is driven by the bellow (or pumping) action, which removes both heat and moisture from the footwear microclimate^18,19^. The first paper to explore footwear ventilation was probably published by Koch and Kaplan in 1956^20^, who used carbon dioxide as a tracer gas and applied a gas dilution method for determining the air exchange rate of the toe-cap. The volume of the footwear space, however, also needs to be determined before the ventilation rate is calculated. Satsumoto et al.^18^ applied a steady-state tracer gas method for directly measuring the ventilation rate using carbon dioxide. The results provided reasonable comparative estimates of the ventilation rate at different footwear locations, but might not be sufficiently accurate because the penetrating tubes would have alleviated the bellow action of the shod foot. Moreover, the diffusivity of carbon dioxide is smaller than that of water vapor, and a correction is required if the ventilation is poor^21^.

In this study, we aimed to achieve the following: (1) develop a new method for investigating the spatial characteristics of the footwear microclimate with a minimal effect on foot motion and without direct contact with the foot skin; (2) develop a new method for measuring the ventilation rate inside footwear; and (3) investigate the relationship between these measured characteristics of the footwear microclimate and the microbial community populations and diversity of the foot plantar skin. Three types of footwear were tested, casual shoes, running shoes, and perforated shoes, and pairwise comparison was performed between the three types in terms of the footwear microclimate and corresponding foot microbial community.

## Methods

### Participants and test footwear

Three healthy Chinese men (age: 28.3 ± 2.3 years; height: 175.3 ± 2.5 cm; body mass: 65.3 ± 2.3 kg) from the same office with a regular daily routine (at least a 5-h continuous stay in the office from 1 pm) were recruited. All three participants were physically active and free of lower extremity injury or pain, and no foot infections were reported. The experimental procedures were explained to the participants before obtaining written informed consent. Our experiment was approved by the Human Research Ethics Committee of the University of Hong Kong. All methods were performed in accordance with the relevant guidelines and regulations.

To evaluate the influence of footwear types on the microclimate formation inside footwear, three kinds of footwear with different upper materials were tested, including casual shoes with a suede upper, running shoes with a textile upper, and perforated shoes with a rubber upper with a dense array of holes on the surface (Fig. S1). The water vapor transmission rate (WVTR) of the upper materials was tested according to the ASTM E96 Standard^22^ (Table S1). All footwear was custom-fitted to each participant’s foot size because the fitting factor was not considered in this study, although it is known to have an effect on the formation of the footwear microclimate^18^. All of the original insoles were replaced by uniform ultraviolet (UV)-sterilized insoles to eliminate the effect of insole variation on the formation of the internal microclimate, as well as to prevent those insoles from contaminating the foot plantar skin.

### Experimental procedures

**Figure 1:**
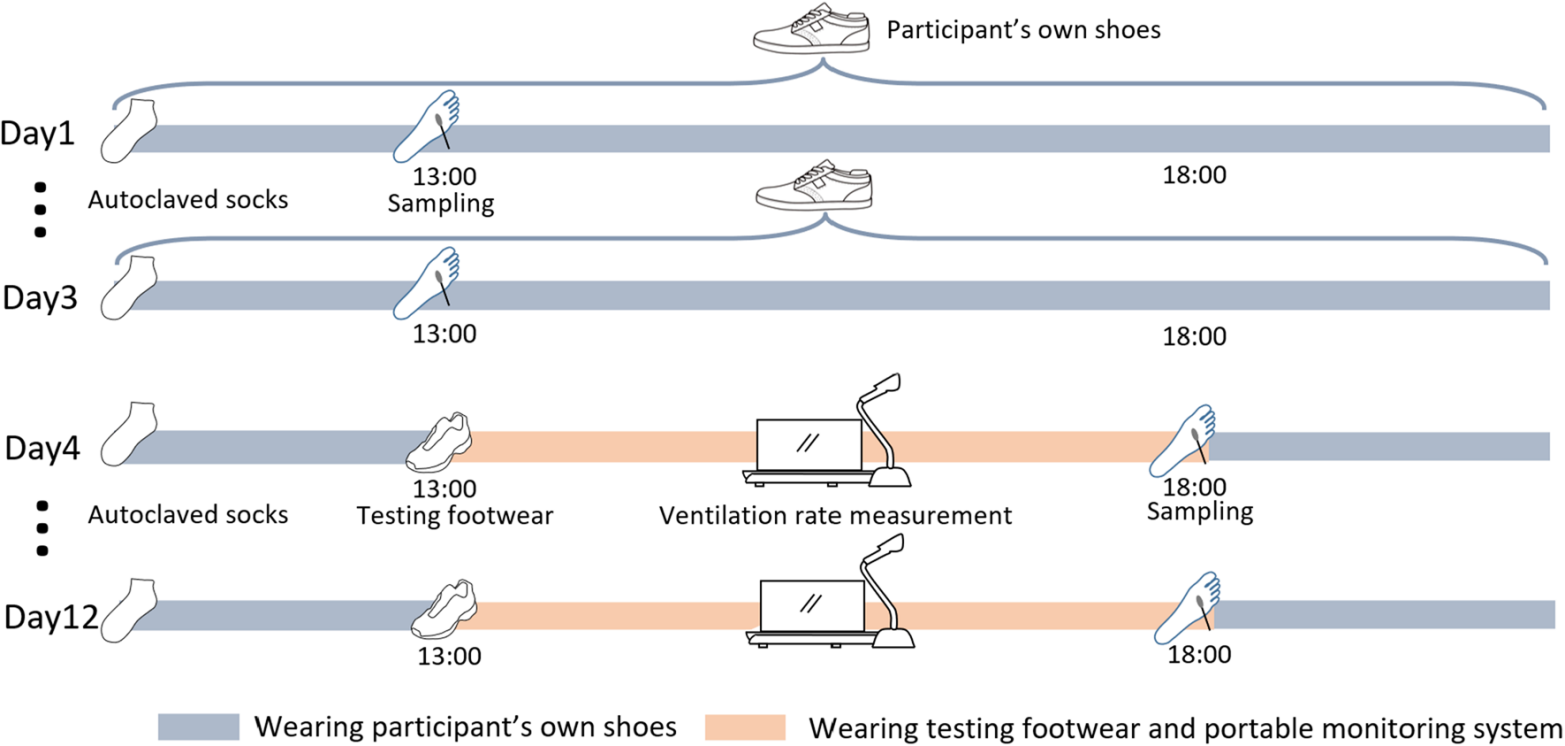
Schematic of procedures for the whole 12-continuous-day experiment (Day 1–Day 3: wearing the participant’s own shoes; Day 4–Day 12: wearing each testing footwear for three consecutive days). The testing order of the three footwear types was balanced (Participant A: 1. perforated shoes; 2. casual shoes; 3. running shoes; Participant B: 1. running shoes; 2. perforated shoes; 3. casual shoes; Participant C: 1. casual shoes; 2. running shoes; 3. perforated shoes).

The experiment lasted 12 continuous days, as illustrated in Fig. 1, from March 14 to 25, 2020, during which all three participants were asked to maintain their daily routine, including office hours and bathing time. The participants wore the same regular shoes before switching to the testing footwear to guarantee a relatively stable condition of the microbial community at the plantar skin at the beginning of the experiment. Autoclaved socks were provided to the participants every day for the same purpose. The first 3 days served as a control group to establish the initial microbial concentration and community before switching to the testing footwear. Because three kinds of footwear were tested and each was tested three times in succession, each participant conducted a total of nine trials in the following 9 days, and the testing order of footwear was balanced.

**Figure 2:**
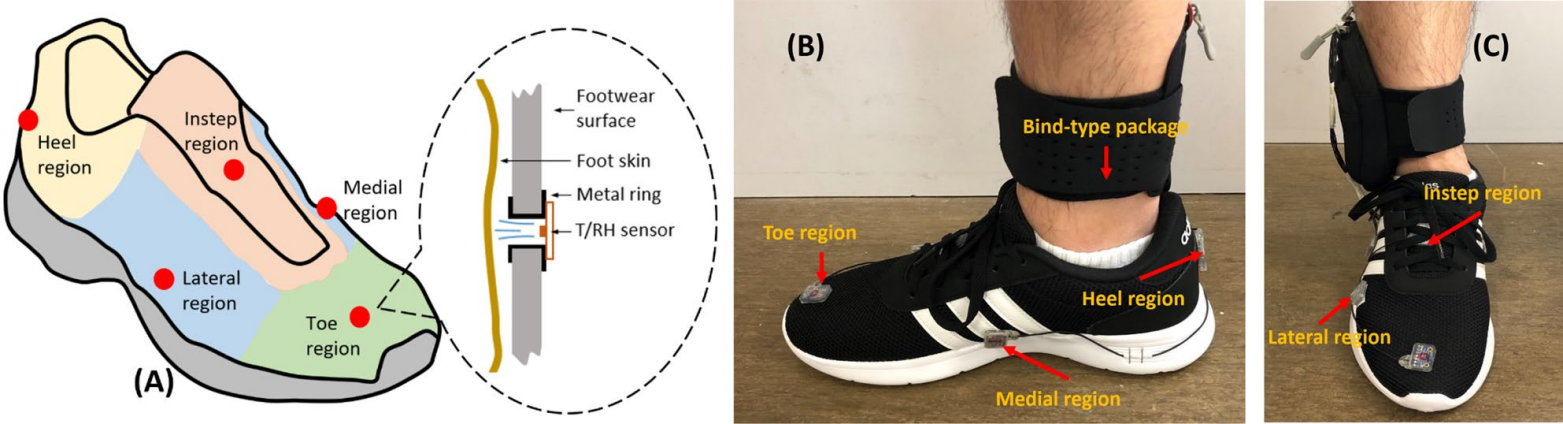
**(A)** shows the five test regions on the right footwear including the toe, medial, lateral, instep, and heel regions. The red dots represent the center of each region where the temperature/relative humidity (T/RH) sensor was installed. The schematic diagram inside the dotted circle shows the air channel created by a metal ring (inner diameter = 5 mm, outer diameter = 10 mm), which allows the T/RH sensor to monitor the footwear microclimate without disturbance of foot motion. **(B,C)** are the side and front view of the portable monitoring system placed on one of the participants.

The spatial characteristics of the footwear microclimate were monitored with a portable system (details in “Portable microclimate monitoring system”) worn by each participant, which did not interfere unacceptably with the foot motions. Thermal data at five regions of the footwear (toe region, medial region, lateral region, instep region, and heel region) were thus obtained and compared (Fig. 2A). A chamber method (details in “Ventilation rate measurement”) was developed for measuring the ventilation rate inside the footwear at gait speeds of 3 km/h and 6 km/h, without removing the portable system, to obtain the water vapor concentration inside the footwear. At the end of the working day, at 6 pm, swab sampling (details in “Plantar skin microbe sampling, DNA extraction, quantification, and sequencing”) was performed on the participants’ distal plantar skin (metatarsal and toe-web regions) and proximal plantar skin (heel region) separately after 5-h continuous wearing of each footwear type. In total, 78 samples were obtained for downstream processing, including 72 (3 participants × 12 days × 2 sites) skin samples and 6 blank samples (2 from negative swabs, 2 from autoclaved socks, and 2 from UV-sterilized insoles).

The temperature and humidity of the office were stable at 23.4 ± 0.2 °C and 79.3 ± 3.0% respectively during the experiment.

### Portable microclimate monitoring system

To measure the temperature and humidity of the microclimate between foot and footwear, circular holes edged by metal rings (inner/outer diameter = 5/10 mm) were manufactured and distributed on the footwear surface at five positions to represent the five regions listed above, creating an air channel in the microclimate. Integrated temperature and humidity sensors (SHT31, Sensirion, Staefa, Switzerland) were attached on the outer side of the circular holes with the probes facing inside (Fig. 2A). The in-shoe temperature and humidity at the five regions could therefore be measured objectively due to the created air channel, and less discomfort was caused for the wearer because the sensors did not occupy the narrow space inside the footwear. The SHT31 sensors were calibrated using a calibrated thermostatic chamber (SM-105-CB-WT, Sanwood, Guangdong, China). An assembled controller (Adafruit Metro 328, Adafruit, New York, NY, USA) was used to establish the monitoring frequency of 0.1 Hz with a real-time clock function. Nine-volt rechargeable lithium batteries were used to enable 5-h continuous monitoring. The controller and battery were placed in a bind-type package that could be tied to the participant’s calf. The whole portable system weighed 142.4 ± 3.2 g and was user-friendly with no effect on the participant’s movement (Fig. 2B,C). Only the footwear microclimate of the right foot was measured under the assumption of symmetry.

### Ventilation rate measurement

**Figure 3:**
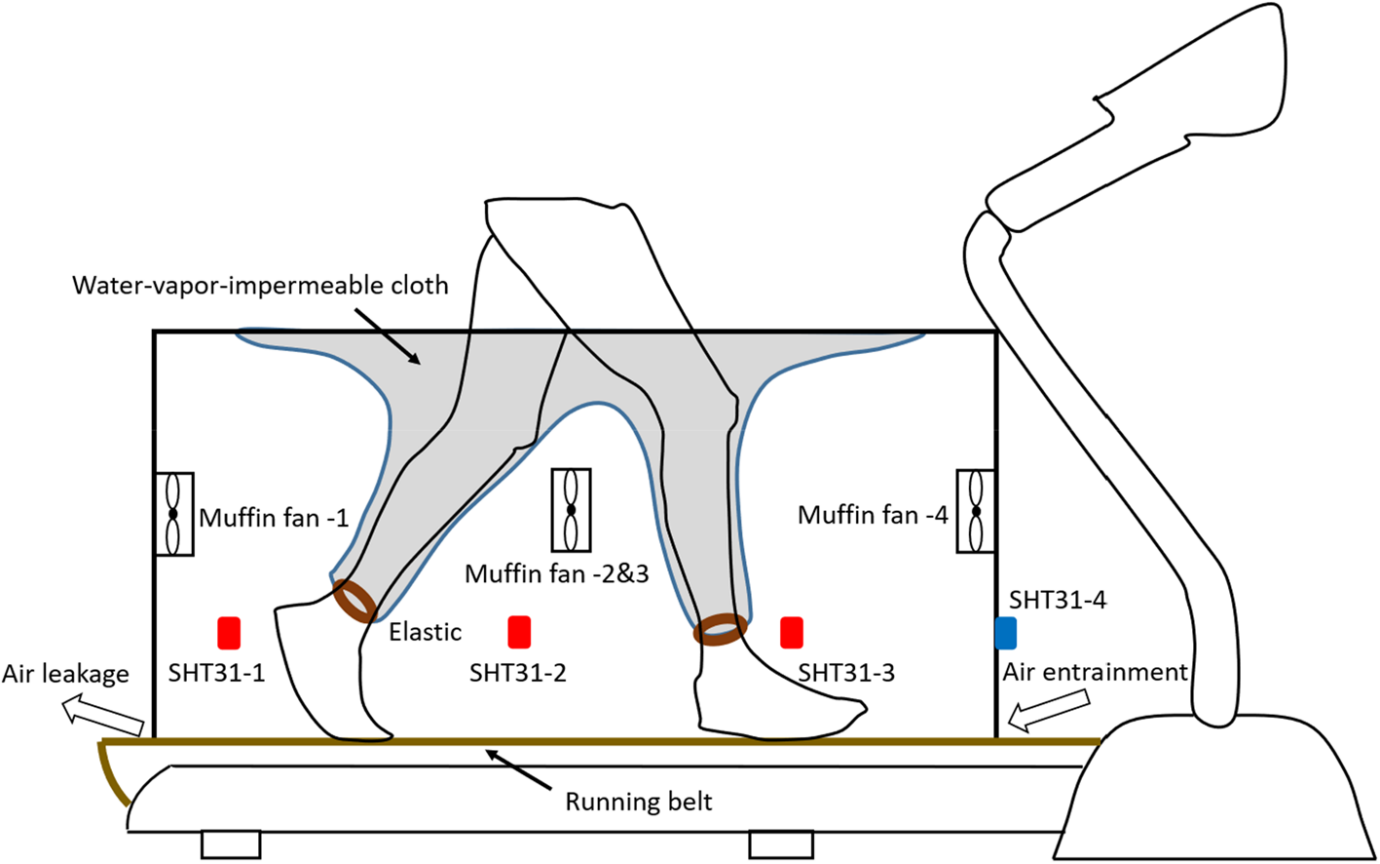
Schematic diagram of the chamber setup for measuring the footwear ventilation rate.

A chamber method was developed for evaluating the ventilation rate inside the footwear, in which water vapor was considered as the tracer gas. Figure 3 shows the schematic diagram of the system setup, where an acrylic chamber (1050 mm × 580 mm × 605 mm) was placed on a treadmill to create a relatively isolated space. Four small muffin fans (11 L/s, AFB0612H, Delta Electronics Inc., Taiwan) were placed at each side to ensure that the air was well-mixed inside the chamber. Three SHT31 sensors were installed at the front, middle, and back of the chamber’s interior, and one was installed outside the chamber near the running belt inlet for measuring the temperature and humidity of air brought into the chamber. A piece of water–vapor-impermeable cloth was placed on top of the chamber together with two legs, which performed as a pair of pants to allow the participants to move freely on the treadmill as well as to avoid the effect of sweat evaporation from participants’ legs to the chamber environment. Equation (1) represented the concentration change of water vapor inside the chamber, in which the footwear was considered as the only internal source of moisture. The ventilation rate ${Q}_{f}$ inside the footwear could therefore be calculated under the steady-state condition as shown in Eq. (2).

1 $${V}_{c}\cdot \frac{d{M}_{c}}{dt}=2{Q}_{f}\cdot \left({M}_{f}-{M}_{c}\right)+{Q}_{c}\cdot \left({M}_{r}-{M}_{c}\right)$$

2 $${Q}_{f}={Q}_{c}\frac{{M}_{c}-{M}_{r}}{2\left({M}_{f}-{M}_{c}\right)}$$

where ${V}_{c}$ is the volume of the chamber (L), ${Q}_{c}$ (L/min) is the ventilation rate of the chamber at different treadmill speed modes, which was measured using the tracer gas method (Fig. S2), ${M}_{c}$ and ${M}_{f}$ (g/m^3^) are the averaged water vapor concentrations of the air inside the chamber and the footwear, respectively, under steady conditions, and ${M}_{r}$(g/m^3^) is the water vapor concentration of the air flowing into the chamber from the room environment. The “2” represents the fact that the left foot had the same ventilation rate as the right foot based on the assumption of symmetry. The water vapor concentration can be calculated from the temperature $T$ (K) and relative humidity $RH$ (%) using Tetens’s equation^23,24^:

3 $$M=0.61078exp\left(\frac{17.27T}{T+237.3}\right)\frac{21.674\times RH}{\left(T+273.15\right)}.$$

The measurement was performed in an environmental chamber, which provided a relatively constant air temperature of 22.0 ± 0.2 °C, a relative humidity of 76.2 ± 2.8%, no solar radiation, and a low air movement. Two gait speeds were selected for the testing, 3.0 and 6.0 km/h, to represent walking and jogging. Because three types of footwear were tested, and each type was tested three times, a total of nine trials were carried out for each participant. Prior to each trial, the participants were instructed with regards to the testing protocol: walking on the treadmill for 20 min at 3 km/h, resting for 10 min, jogging on the treadmill for 15 min at 6 km/h, and resting for 5 min before finishing the testing. Figure S3 showed an example of the change in water vapor concentration inside the footwear, inside the chamber, and outside the chamber during the 50 min. The concentration values under steady-state were obtained by applying the ExpDecay3 fitting function on the scatter diagram in Origin 2018 (OriginLab, USA), and the derivation process is shown under the Figure S3 in the supporting document.

The chamber method was calibrated using a sealed acrylic box (100 mm × 100 mm × 280 mm) with two inlets and two outlets, which was put inside the chamber to perform as the “shod feet” (details in Figure S4). Table S2 showed the results of the calibration experiment, which were then applied on the calculated ventilation rates obtained from the participant involved experiments. Here, the calculated ventilation rate means the total ventilation rate of the two shoes.

### Plantar skin microbe sampling, DNA extraction, quantification, and sequencing

To investigate the relationship between the footwear microclimate and the microbial community of the foot plantar skin, swab sampling was performed at the distal plantar skin (metatarsal and toe-web regions) and the proximal plantar skin (heel region) separately. Autoclaved double swabs, pre-moistened with PBST solution (0.15 M NaCl with 0.1% Tween 20)^25^, were rubbed back and forth along the skin surface. Each surface was sampled for 30 s covering the whole corresponding areas (avoiding the toenails). Negative swab samples taken without touching the foot skin surfaces were included as a control. Samples from autoclaved socks and UV-sterilized insoles were also included. The samples were subsequently stored at − 20 °C within 30 min of sampling and until DNA extraction. Genomic DNA (g-DNA) was extracted using a DNeasy PowerSoil Kit (Qiagen, Venlo, The Netherlands), and was dissolved in the Tris–HCl solution for downstream applications.

The quantification of bacterial 16S rRNA-encoding was completed using real-time quantitative polymerase chain reaction (qPCR), which was performed in triplicate using a CFX Connext real-time PCR system (Bio-Rad Laboratories Inc., CA, USA). Universal bacterial primers and probes covered the 331 to 797 *E. coli* numbering region of the 16S rRNA-encoding gene with forward primer 5′-TCCTACGGGAGGCAGCAGT-3′, reverse primer 5′GGACTACCAGGGTATCTAATCCTGTT-3′, and probe (6-FAM)-5′-TATTACCGCGGCTGCTGGCAC-3′-BHQ-1)^26^. The qPCR reaction was performed in a total volume of 20 µL using PrimeTime Gene Expression Master Mix (Integrated DNA Technologies, Inc., IA, USA), containing 500 nM of each of the forward and reverse primers, 250 nM of probe, and 1 µL of DNA template. The reaction conditions for amplification of DNA were 95 °C for 3 min, and 50 cycles of 95 °C for 15 s and 60 °C for 1 min. Real-time qPCR standard curves of genome quantity vs. cycle threshold number for bacteria were developed using known amounts of *Bacillus subtilis* (ATCC 6633) genomic DNA. Three independent tenfold dilution series were produced corresponding to 10^0^ to 10^6^ genome copies of *B. subtilis*, revealing the qPCR efficiency to be 84.8%. The unit for the calculated bacterial concentration was log_10_(operon copies) per µL DNA template when regarding the number of 16S rRNA operon copies per genome of *B. subtilis* to be 10^27^. Besides the bacterial concentration data, we also calculated the bacterial increase ratio after 5-h use of three footwear types to represent the bacterial growth during the experiment. The data were obtained by dividing the initial bacterial concentration (operon copies per µL) into the final bacterial concentration (operon copies per µL). Here, the initial bacterial concentration data were obtained by averaging the concentration data of the first 3 days under the assumption that the initial microbial condition at the plantar skin remained consistent for the following 9-day experiments.

The sequencing of the bacterial 16S rRNA V3–V4 region was conducted by Novogene (Beijing, China), who assisted with library preparation and microbial amplicon sequencing on an Illumina PE250. The workflow for the DNA sequencing including the PCR amplification, PCR products purification, library preparation and sequencing. To guarantee the reliability of the data, quantity control (QC) was performed at each step of the procedure using the Nanodrop for the tests of DNA purity (OD260/OD280) and the agarose gel electrophoresis for the tests of DNA degradation and potential contamination. The PCR amplification was performed by the primers 341F and 806R, which were based on the V3-V4 hypervariable region of prokaryotic 16S rDNA for the detection of bacteria. Libraries were performed on a paired-end Illumina PE250 platform to generate 250 bp paired-end raw reads. The paired-end reads were assigned to samples based on their unique barcode and truncated by cutting off the barcode and primer sequence. Quality filtering on the raw reads was performed under specific filtering conditions to obtain the high-quality clean reads.

The QIIME2 (v.2019.10) was used to process the raw sequences and sequence quality filtering was performed with the DADA2 algorithm for detecting and correcting Illumina amplicon sequence data^28^. First, the “import” command was applied to de-multiplex and import the raw paired-end fastq files into QIIME2. The “data2 denoise-paired” plug-in package was then used to execute the DADA2 algorithm to generate amplicon sequence variants (ASVs). The imported paired-end sequences were filtered, trimmed, de-noised, and de-replicated based on a minimum read length of 262 bq. Chimeric and singleton sequences were also removed. A total of 6,360,297 high-quality reads were retained after quality control (~ 67% of raw reads). High-quality sequences were clustered into operational taxonomic units (OTUs) against the Silva_132 rRNA gene sequence database with a 99% sequence identity cutoff^29,30^. The R package (4.0.2) was applied to conduct secondary quality filtering on the identified sequences to remove those (1) with order level unidentified, (2) with order level identified to be Chloroplast, (3) with family level identified to be Mitochondria, and (4) with identity confidence less than 0.95. A total of 4,993,769 reads were retained after the second quality control. The “core-metrics-phylogenetic” command was used to generate the β-diversity metrics, and to generate principal coordinate analysis (PCoA). The pairwise weighted (community structure) and unweighted (community membership) UniFrac distances between each pair of samples were calculated, and the resulting distance matrices were used for all downstream statistical tests of sample similarity and plotted for PCoA.

### Statistical analysis

The obtained data were classified into three groups: thermal, ventilation, and microbial. The thermal data includes time-averaged in-shoe temperature and time-averaged in-shoe absolute humidity with three corresponding independent variables of individual, footwear type, and measuring region. The ventilation data was footwear ventilation rate with three corresponding independent variables of individual, footwear type, and gait speed. The microbial data includes bacterial population, bacterial increase ratio, and bacterial diversity with three corresponding independent variables of individual, footwear type, and skin site. For the bacterial population data, all the qPCR results were presented as the means of the log_10_ value. For all the dependent variables above (expect for the bacterial diversity which was analyzed separately), a three-way repeated measures analysis of variance (ANOVA) was first performed to determine whether there was an interaction effect between their corresponding three independent variables. If no three-way interaction existed, a two-way ANOVA was performed between two of the three independent variables other than the individual since the individual difference was not supposed to be discussed in this article. If two-way interaction was observed, post hoc multiple comparisons with Bonferroni correction were performed on the dependent variable under the interaction of the two corresponding independent variables, and the effects of individual differences on the comparison results could therefore be eliminated. Tests of normality for dependent variables of each group were performed before the comparisons and the results were shown in Table S7. Based on the Shapiro–Wilk test results, the variables of each group satisfied the normal distribution and thus the comparison results made sense. For the bacterial β-diversity analysis, the significance of sample groupings was assessed using permutational ANOVA (PERMANOVA) performed in QIIME, and statistical significance was calculated by comparing the pseudo-F statistic to a distribution generated by 999 permutations of the randomized dataset.

Regression analyses were performed to explore the linear relationship between the microclimate characteristics (temperature, absolute humidity, and ventilation rate) put on the x-axis and bacterial increase ratio put on the y-axis. The bacterial increase ratio was used rather than the bacterial population because the former revealed the bacterial growth during the 5-h use of the testing footwear, while the latter actually represented the bacterial growth under the use of regular shoes and testing footwear successively. When exploring the linear relationship between two dependent variables, their corresponding independent variables were supposed to be consistent. For all the four dependent variables, two common independent variables could be observed, i.e., individual and footwear type, and one was unique. For the dependent variables of temperature and humidity, the one particular independent variable was the measuring region. Therefore, we averaged the data with between one and all five of the regions involved and obtained 31 sets of data for both temperature and humidity ($${C}_{5}^{1}+{C}_{5}^{2}+{C}_{5}^{3}+{C}_{5}^{4}+{C}_{5}^{5}=31)$$. For the ventilation rate, the particular independent variable was gait speed (3 km/h and 6 km/h), and 2 sets of data existed. For the bacterial increase ratio, the particular independent variable was the skin site (distal and proximal plantar skins), and 2 sets of data were obtained as well. Therefore, there were 31 + 31 + 2 = 64 sets of data put on the x-axis, and 2 sets put on the y-axis, and a total of 128 pairs of the linear relationship were analyzed. Statistical analysis was performed using SPSS Statistics V.9.0 (IBM, USA), and the level of significance adopted was 0.05.

## Results

### Statistical results

We could observe in Table S3 that for all dependent variables (except for the bacterial diversity which was analyzed separately), no three-way interaction existed between their corresponding three independent variables, which means the effects of the individual difference can be eliminated when analyzing the two-way interaction between the other two independent variables. For the dependent variables of temperature, absolute humidity, ventilation rate, bacterial population, and bacterial increase ratio, two-way interaction existed between their corresponding independent variables (2) & (3) as shown in the Table S3. The statistical comparisons on the five dependent variables were analyzed separately in the following sections.

### Spatial characteristics of footwear microclimate for three footwear types

**Figure 4:**
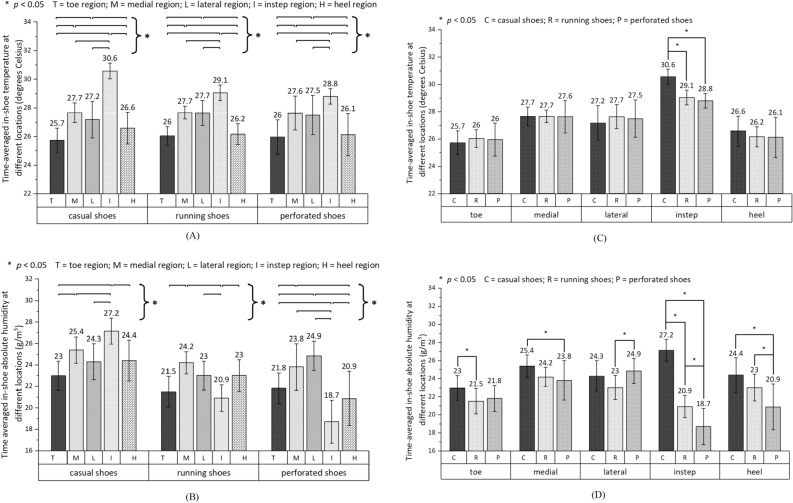
Time-averaged in-shoe temperature (mean ± SD) and absolute humidity (mean ± SD) at five footwear regions for three footwear types separately. **(A,B)** Compare the thermal characteristics between measuring regions, while **(C,D)** compare footwear types. Significant differences (*p* < 0.05) between pairs of bars are indicated by an asterisk.

Figure 4A,B show the time-averaged in-shoe temperature and absolute humidity at the five regions for the three footwear types separately. With statistical significance, the instep region had the highest in-shoe temperature of any region for all footwear types (28.8 ± 0.5 °C to 30.6 ± 0.5 °C). The in-shoe temperatures at the medial and lateral regions were similar to each other (27.6 ± 1.2 °C to 27.7 ± 0.7 °C and 27.2 ± 1.3 °C to 27.7 ± 0.9 °C respectively) followed by the toe region (25.7 ± 0.9 °C to 26.0 ± 1.2 °C) and the heel region (26.1 ± 1.5 °C to 26.6 ± 1.1 °C) for all footwear types. As for the absolute humidity, the instep region had the highest level of moisture content of any region for the casual shoes (27.2 ± 1.2 g/m^3^), but the lowest for the running shoes (20.9 ± 1.2 g/m^3^) and perforated shoes (18.7 ± 2.0 g/m^3^). For all footwear types, the medial region (23.8 ± 2.2 g/m^3^ to 25.4 ± 1.2 g/m^3^) had a similar level of moisture content to the lateral region (23.0 ± 1.3 g/m^3^ to 24.9 ± 1.4 g/m^3^), but a significantly higher level of moisture content than the toe region (21.5 ± 1.4 g/m^3^ to 23.0 ± 1.4 g/m^3^). As for the heel region, its moisture level was similar to the medial and lateral regions for the casual shoes (24.4 ± 1.9 g/m^3^) and running shoes (23.0 ± 1.5 g/m^3^), but was significantly lower than the two regions for the perforated shoes (20.9 ± 2.5 g/m^3^).

When comparing the thermal characteristics between the three footwear types at one measuring region (Fig. 4C,D), no significant difference was observed in the in-shoe temperature at most regions, except for the instep region, where the casual shoes obtained the significantly highest temperature (30.6 ± 0.5 °C). As for the absolute humidity, the casual shoes had the highest moisture level at most regions, except for the lateral region, where the perforated shoes had the highest level of moisture content (24.9 ± 1.4 g/m^3^), although the perforated shoes had the lowest level of moisture content at all other regions.

### Ventilation rates inside footwear at two gait speeds for three footwear types

**Figure 5:**
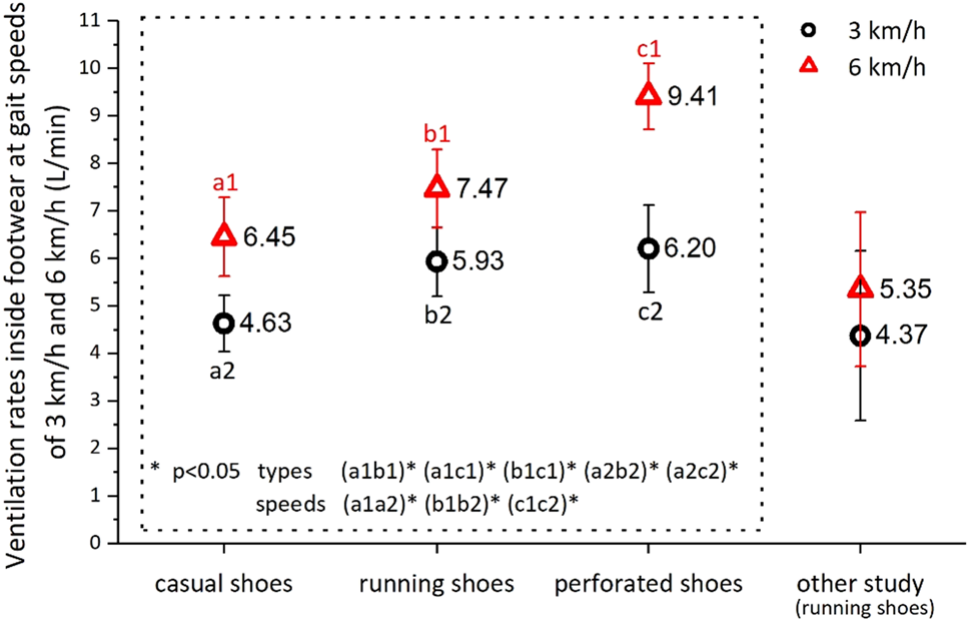
Ventilation rates inside footwear at gait speeds of 3 km/h and 6 km/h for three footwear types separately (Mean ± SD). Results of the “other study” refer to Yasuhiro et al. (2016)^14^.

The ventilation rate inside the footwear was measured at two gait speeds, 3 km/h and 6 km/h, for the three footwear types. Because the measurement was conducted three times for each type for the three participants, a total of 26 sets of data were obtained, with 1 set lost. Figure 5 compares the ventilation rates between the three footwear types as well as between the two gait speeds. The perforated shoes had the highest ventilation rates at 6 km/h (9.41 ± 0.69 L/min), followed by the running shoes (7.47 ± 0.82 L/min). However, no significant difference was observed between the two types at 3 km/h. The casual shoes had the lowest ventilation rates for both gait speeds (6.45 ± 0.83 L/min at 6 km/h and 4.63 ± 0.60 L/min at 3 km/h). Evidently, higher gait speed resulted in a larger ventilation rate for all three footwear types. The ventilation rates were higher than those measured in previous studies for the same footwear types^14^.

### Microbial quantitative results at the distal and proximal plantar skins after 5-h use of the three footwear types

**Figure 6:**
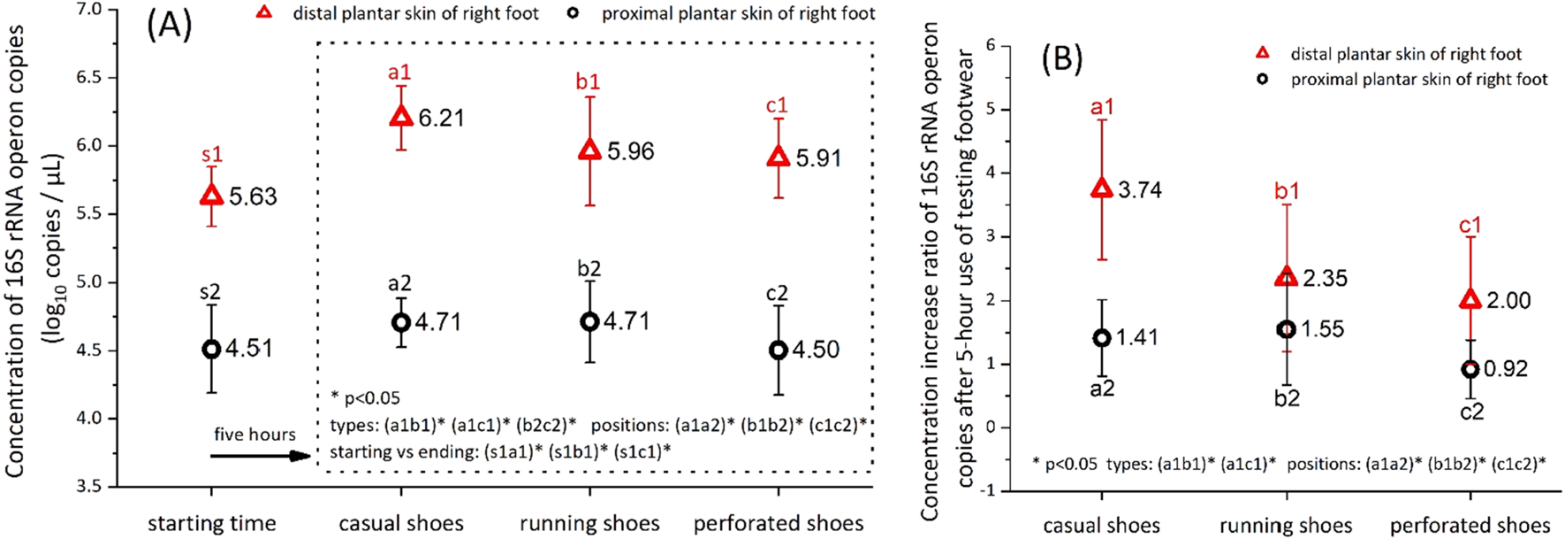
Concentrations **(A)** and concentration increase ratios **(B)** of 16S rRNA operon copies at the distal plantar skin (metatarsal and toe-web regions) and the proximal plantar skin (heel regions) of the right foot after 5-h use of three footwear types (mean ± SD).

Figure 6A shows the concentrations of 16S rRNA operon copies at the distal plantar skin (metatarsal and toe-web regions) and the proximal plantar skin (heel region) of the right foot before and after 5-h use of the three types of footwear. The bacterial population at the distal plantar skin was significantly higher than that at the proximal skin for all footwear types. We also observed that the bacterial population at the distal plantar skin increased with time when enveloped by the footwear, while no significant increase was observed at the proximal plantar skin after the same duration. When enclosed with the casual shoes, the bacterial population on the distal skin increased to 6.21 ± 0.23 log_10_/µL, which was significantly larger than that with running shoes and perforated shoes (5.96 ± 0.40 log_10_/µL and 5.91 ± 0.29 log_10_/µL respectively). However, no significant difference was observed in the proximal skin bacterial population when enclosed by the casual shoes or the running shoes for 5 h, although a significant difference in the value could be seen between the running shoes and perforated shoes (4.71 ± 0.30 log_10_/µL and 4.50 ± 0.33 log_10_/µL respectively).

Figure 6B shows the concentration increase ratios of 16S rRNA operon copies at the plantar skin after 5-h use of the three footwear types, which can be understood as the bacterial growth. In agreement with the results in Fig. 6A, the bacterial increase ratio at the distal plantar skin was significantly higher than that at the proximal plantar skin for all footwear types. The comparison results between footwear types were consistent with the results in Fig. 6A, except for the proximal plantar skin where no significant difference was observed in the bacterial increase ratio between all footwear types.

### Microbial diversity of the distal and proximal plantar skins after 5-h use of the three types of footwear

**Figure 7:**
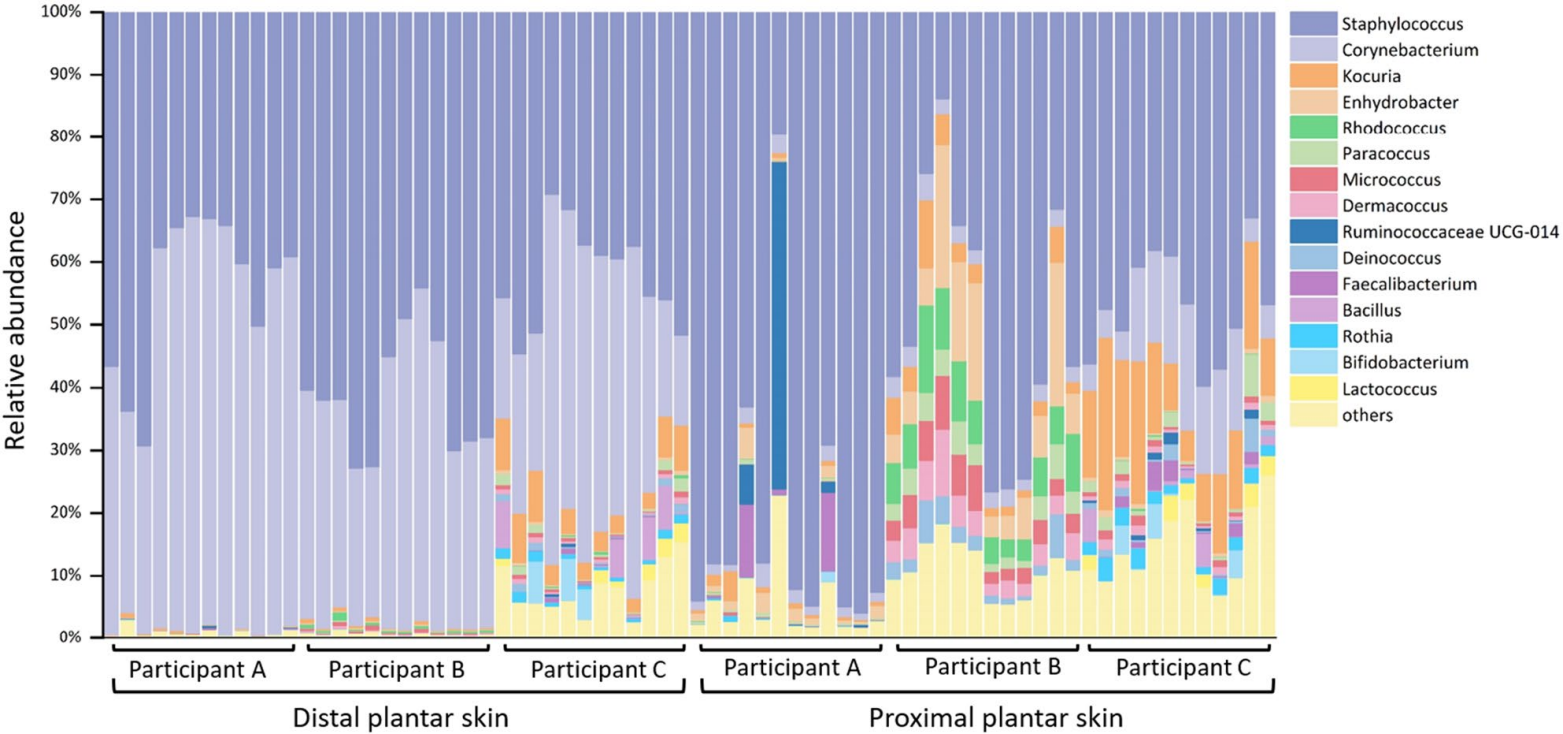
Variation of bacterial community composition between participants for the 15 most dominant genera observed across the distal and proximal plantar skins.

Among the 72 swabbing samples, a total of 4,993,769 sequences passing quality control were clustered into 1238 distinct OTUs, the majority (72.3%) of which were identified at the genus level. No sequences were obtained from the negative controls (unused swabs, socks, and insoles) as insufficient DNA was generated in the amplicon sequencing process. Common skin commensals including *Staphylococcus* (average relative abundance of 54.46%), *Corynebacterium* (23.69%), *Kocuria* (4.00%), and *Enhydrobacter* spp. (2.16%) were present in all plantar skin samples (Fig. 7)^31–34^. The figure also indicates an increase in the bacterial diversity of the proximal plantar skin compared with the homogenous nature of the distal skin. The differences in diversity could also be observed in the β-diversity results, which revealed significant clustering based on individuals and skin sites. The clustering of individuals was demonstrated to be significant by the unweighted UniFrac distances between samples (Fig. S5A), while the significant clustering of skin sites was clearly demonstrated by both the weighted and unweighted UniFrac distances (Fig. S5B and S5D). However, no significant clustering could be observed between footwear types in either community membership or community structure (*p* = 0.723, and *p* = 0.495, respectively, from Table S4). To eliminate the effects of individuals and skin sites on the diversity analysis of the footwear types, we divided the samples into six subgroups (3 participants × 2 skin sites) and compared the weighted/unweighted UniFrac distances between footwear types within each subgroup. Significant differences in weighted UniFrac distance were observed between the footwear types at the distal plantar skin for all participants (*p* = 0.006, *p* = 0.004, and *p* = 0.003, respectively, for the three participants) (Table S5). One participant had significantly different bacterial communities at the proximal plantar skin in both weighted and unweighted Unifrac distances with different footwear types (*p* = 0.015 and *p* = 0.043 respectively). Here, we need to point out that the bacterial diversity at the plantar skin obtained at the end of the experiment revealed the microbial communities under the use of the regular shoes and the testing footwear successively.

### Relationship between footwear microclimate and plantar skin bacterial growth

**Figure 8:**
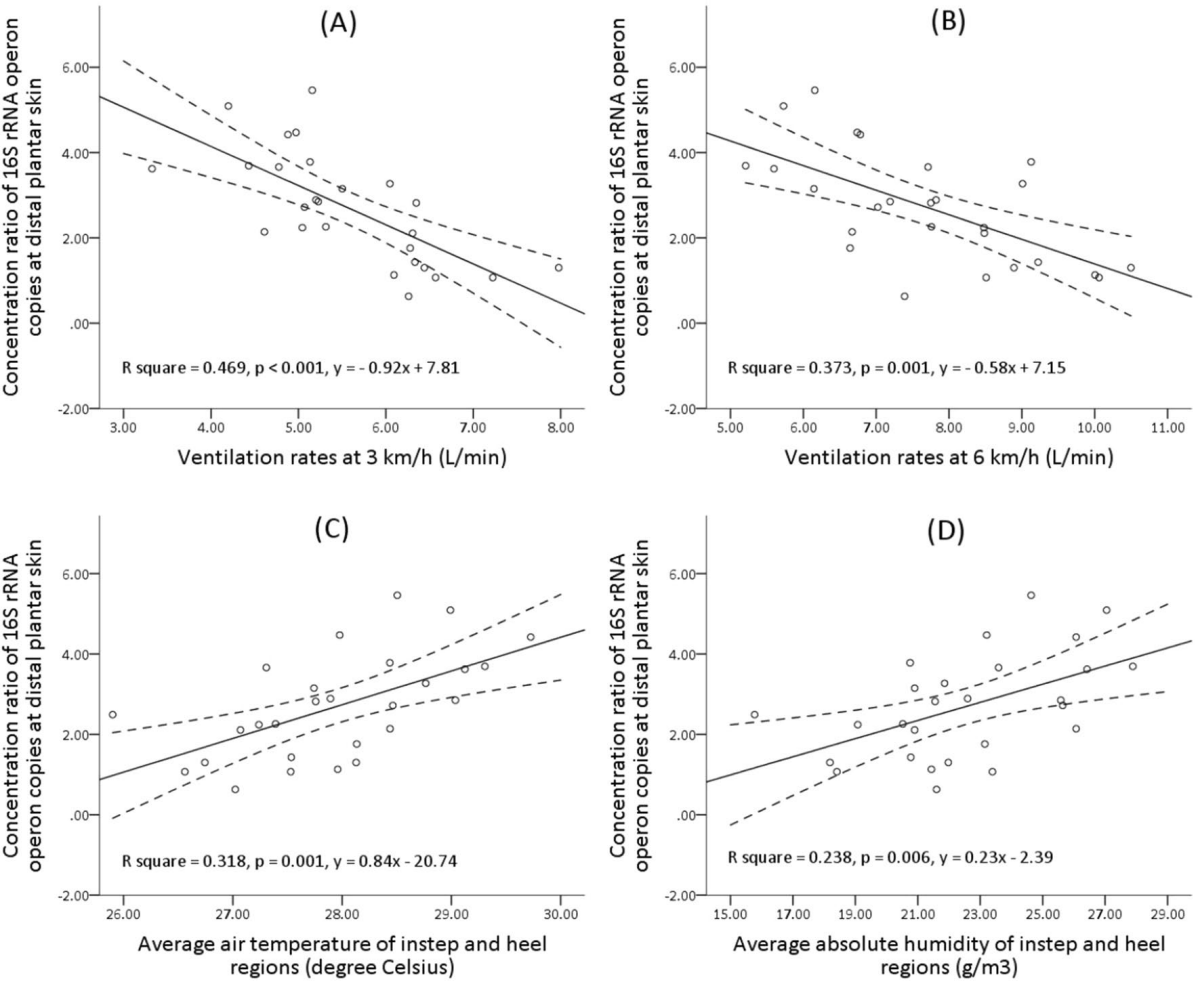
Linear regression results (scatters and fit lines) between footwear microclimate [ventilation rate at 3 km/h **(A)**, ventilation rate at 6 km/h **(B)**, average in-shoe temperature of instep and heel regions **(C)**, and average absolute humidity of instep and heel regions **(D)**] and bacterial growth rate at the distal plantar skin of the right foot.

Regression analyses were performed to explore the linear relationship between the footwear microclimate characteristics (in-shoe temperature, absolute humidity, and ventilation rate) and microbial quantitative data (bacterial increase ratio) at the distal and proximal plantar skins. A total of 128 pairs of regression results (adjusted R^2^ and significance) are shown in Table S6. No significant linear correlation was observed between the bacterial increase ratio at the proximal plantar skin and any of the microclimate characteristics, while significant linear correlations could be found between that at the distal plantar skin and the microclimate characteristics. The ventilation rate at 3 km/h showed a more significant linear correlation (R^2^ = 0.469, *p* < 0.001) with the bacterial increase ratio than at 6 km/h (R^2^ = 0.373, *p* = 0.001), and these negative linear correlations can be seen in Fig. 8A,B. As for the temperature, few of the average values showed significant positive linear correlations with the bacterial increase ratio, and the most significant correlation was observed for the average values of the instep and heel regions as shown in Fig. 8C (R^2^ = 0.318, *p* = 0.001). As for the absolute humidity, most of the average values showed significant positive linear correlations with the bacterial increase ratio, and the average values of the instep and heel regions also showed the most significant correlation with the bacterial increase ratio (R^2^ = 0.238, *p* = 0.006). The positive linear correlation can be seen in Fig. 8D.

## Discussion

### Footwear spatial microclimates and footwear types

In this study, the microclimates at five footwear regions were measured and compared, including the toe, medial, lateral, instep, and heel regions. The microclimate was investigated regionally for three reasons: (1) physiological characteristics vary at different foot skin regions due to the distributions of sweat glands and blood flow, which contribute to variations in heat and moisture generation at the skin surfaces^3,35–37^; (2) thermal properties (insulation and vapor resistance) and physical properties (air permeability and water absorption) vary at different surface regions due to different materials and constructions of footwear, which contribute to variations in heat and moisture conservation and dissipation^38–41^; and (3) air layer thickness and air movement between foot and footwear vary spatially and temporally due to irregular foot shapes and foot motions, which contribute to variations in convective heat and moisture transfer at different regions^18,42^. Therefore, we applied integrated T/RH sensors at five representative footwear regions for 5-h continuous monitoring with minimal effect on the foot motions and corresponding microclimate to investigate the spatial characteristics of the footwear microclimate for three footwear types.

We found that the distributions of the in-shoe temperature at the five regions were consistent with the temperature distributions of the foot upper skin measured in Shen W. W. et al.’s study^43^, with the instep region sustaining the highest temperature, followed by the medial and lateral regions, and then the heel and toe regions for all footwear types. The in-shoe temperature distributions also agreed with the results in West A. M. et al.’s work ^10^ that the instep region obtained a significantly higher in-shoe temperature than the toe region for shoes with high air permeability, although the discrepancy narrowed for shoes with low air permeability. As for the humidity, the moisture content distributions at the five regions varied in footwear types, especially for the instep region, which sustained the highest level of moisture content for the casual shoes, but the lowest for the running and perforated shoes. We can explain that for the running and perforated shoes, moisture at the instep region was dissipated due to the higher local ventilation rate between the instep and adjusted tongue in comparison with the other regions^18^. But for the casual shoes, the increase in the water vapor storage capacity due to the high local temperature could compensate for the increase in the moisture dissipation due to the high local ventilation, and also the adjusted tongue of the casual shoes was not as flexible as that of the other two types, which resulted in a higher level of humidity at the instep region. The moisture distributions at the other four regions, however, varied for different footwear types with different upper materials and constructions.

As for the effect of footwear types on microclimate formation, previous studies have demonstrated that footwear with leather uppers accumulated more heat and moisture internally than footwear with mesh spacer fabric uppers^44^. An open upper structure (with holes on the surface) also results in lower temperatures and levels of moisture accumulation than a closed upper structure^10^. However, in our results, no significant difference was observed in the in-shoe temperature at most regions among the three footwear types, although a significant difference could be found at the instep region, where the casual shoes sustained the highest local temperature. As for the absolute humidity, in agreement with previous studies^44^, casual shoes accumulated a higher level of moisture content than the other two types, except in the lateral region. The comparison results in moisture content between the running shoes and perforated shoes varied in measuring regions. It seems that the two types had similar overall internal microclimates when worn by participants, which is inconsistent with our expectations that the perforated shoes should have a better performance on the heat and moisture dissipation.

### Footwear ventilation rates and footwear types

The ventilation rate can be regarded as an indicator of the convective heat/mass transfer and moisture dissipation inside footwear due to the bellow action, which plays an important role in cooling and dehumidifying the footwear microclimate^18^. The mechanism of the bellow action can be understood as the periodic oscillation of the foot inside footwear during gait cycles. When the foot lands on the ground during the stance phase, foot-stamping and deformation of the footwear squeeze the warm and moist air out of the footwear. When the foot leaves the ground during the swing phase, fresh air is drawn in via openings and pores in the footwear. Thus, an airflow circulates inside the footwear and dissipates heat and moisture to the outside during gait cycles. Empirically, the faster the gait, the larger the ventilation rate, which is in agreement with the results obtained. The results also demonstrated that the faster the gait, the larger the discrepancy in the ventilation rate between different footwear types. Among the three footwear types, the casual shoes had the lowest ventilation rates for both gait speeds due to the low water vapor permeability of the suede upper and less flexible adjusted tongue, while the perforated shoes obtained the highest ventilation rates due to the dense distribution of holes on the upper surface at gait speed of 6 km/h.

When comparing the results with previous studies^14^, we found that higher ventilation rates were obtained in this study for the same footwear type at the same gait speed. Our results might be more accurate because of the use of sensors without gas tubes penetrating the footwear, as such tubes interfere with the bellow action.

### Bacterial populations and diversity of foot plantar skin for different footwear types

The human foot provides more favorable conditions for the colonization and growth of bacteria than other parts of the human body, due to its high moisture content and a steady supply of nutrients, as well as optimal temperature distribution when enclosed by footwear^45^. The dense eccrine sweat glands distributed on the foot skin (366 glands/cm^2^ on the plantar skin and 149 glands/cm^2^ on the instep skin)^19^ contribute to the moist internal environment. Here, two regions were selected for a better investigation of the bacterial population and diversity on the foot skin. One was the distal plantar skin region, including the metatarsal and toe-web sites, and the other was the proximal plantar skin region, i.e., the heel site. The former has been documented to sustain the highest microbial populations on the foot^45^, while the latter has been reported to present a complex diversity of the microbial community^46^.

The results showed that the bacterial population and increase ratio at the distal plantar skin was significantly higher than that at the proximal skin, while the bacterial diversity at the distal skin was lower, being dominated by *Staphylococcus* spp. and *Corynebacterium* spp., which was consistent with previous studies^32,47,48^. The bacterial population at the proximal plantar skin was more stable during the same time duration than that at the distal skin when enclosed by the footwear. The casual shoes seemed to provide the most favorable internal environment for bacterial growth at the distal skin, followed by the running shoes and perforated shoes with similar increases in bacterial population to one another. As for the bacterial diversity, however, no significant clustering based on footwear types was observed when comparing either community structure or community membership, although significant clustering was found based on individuals and skin sites. When removing the two dominant factors (individual and skin site) and performing the diversity analysis within each subgroup on footwear types, significant clustering based on bacterial community structure could be observed at the distal plantar skin for all participants, which indicated that the community structure at the distal plantar skin could be affected by the microclimate formed in various footwear types, although the nature of the relationship depended on the individuals.

### Relationship between footwear microclimate and plantar skin bacterial growth

To the best of our knowledge, only one existing study has directly investigated the relationship between the footwear microclimate and foot microbial community, and found that the internal environment of the footwear was a risk factor for tinea pedis according to qualitative results^11^. In this study, we not only measured the in-shoe temperature, absolute humidity, and ventilation rate as characteristics of the footwear microclimate, but also collected the microbial population data from foot plantar skin samples, and explored the relationship quantitatively. We found that the bacterial increase ratio at the distal plantar skin showed a positive linear correlation with the in-shoe temperature and absolute humidity and a negative linear correlation with the ventilation rate, indicating that the increased temperature and humidity inside footwear due to decreased ventilation rate supported the bacterial growth on the foot plantar. No significant linear correlation was observed between those microclimate characteristics and the bacterial increase ratio at the proximal plantar skin because the site sustained a relatively stable bacterial population when enclosed by footwear.

Although significant positive linear correlations were observed between the bacterial increase ratio and the internal temperature and humidity, we need to point out that not all the average values with between one and all five of the regions involved had a significant correlation with the bacterial increase ratio. The correlations are more likely to exist in average humidity values. Of all average values, the one combining instep and heel regions showed the most significant correlations with the bacterial increase ratio for both temperature and humidity. To explain such positive linear correlations, we first need to understand the factors directly affecting the skin bacterial populations are, in fact, the skin temperature and humidity, as confirmed by previous studies indicating that body sites with higher temperature and humidity (i.e., axilla, perineum, and foot) supported higher bacterial populations than less warm and moist sites^49^. Therefore, when investigating the relation between the footwear microclimate and skin bacterial population, we need to understand the relation between the footwear microclimate and skin temperature and humidity. As discussed above, the in-shoe temperature distributions have been shown to be consistent with the temperature distributions of the foot upper skin^43^, which to some extent indicates a positive correlation between in-shoe temperature and skin temperature. This is consistent with the positive linear correlation between in-shoe temperature and skin bacterial increase ratio observed here. As for the humidity, first, we replace the abstract concept of “skin humidity” with a more concrete concept, “skin wetness”, which indicates the concentration of water vapor at the skin surface^50^. If the footwear environment sustains a higher level of absolute humidity with corresponding higher internal water vapor pressure, the evaporative capacity of the skin would become lower and the skin wetness would increase then^51^. Therefore, it is unsurprising that the bacterial growth at the plantar skin was in a positive linear correlation with the internal absolute humidity. Besides the temperature and humidity, the ventilation rate inside footwear seems to be a more reliable indicator of the bacteria growth at the plantar skin based on the correlation results. This might due to the mechanism of the impact of footwear ventilation rate on the bacterial population is not only its effect on convective heat transfer at the skin surface, but also its effect on moisture dissipation from the internal environment.

## Conclusion

To investigate the relationship between the footwear microclimate and the microbial community of the plantar skin, experiments with three participants were conducted using novel methods developed for measuring the in-shoe temperature and absolute humidity locally, as well as the overall ventilation rate inside the footwear. Three types of footwear were tested including casual shoes, running shoes, and perforated shoes for pairwise comparison of footwear microclimate and the corresponding microbial community at the plantar skin.

Significant differences were found between footwear types in terms of local temperature with the casual shoes sustaining the significantly highest temperature at the instep region, although no significant difference was observed at other regions in temperature between footwear types. The instep region also sustained the warmest of all regions for all types, followed by the medial and lateral regions, and then the heel and toe regions, which was consistent with the temperature distributions of the foot upper skin. Significant differences were also observed in local internal absolute humidity between footwear types, with casual shoes having the highest level of humidity in most regions. The ventilation rate increased with an increase in gait speed, and the faster the gait, the larger the discrepancy in ventilation rate between footwear types (perforated shoes > running shoes > casual shoes). The casual shoes also seemed to provide the most favorable internal environment for bacterial growth at the distal plantar skin, and the community structure at the distal plantar skin could be affected by the microclimate of different footwear types to each participant. As for the relation between microclimate and microbial populations, we found that the bacterial growth at the plantar skin showed a positive linear correlation with the in-shoe temperature and absolute humidity, but a negative linear correlation with the ventilation rate. The linear correlation was more significant for the ventilation rate than the temperature and humidity, indicating the ventilation rate is an effective parameter to reveal the footwear microclimate, thus the bacterial growth on the plantar skin.

We have presented the first detailed study combining the measurement of the footwear microclimate temperature and humidity with ventilation rates and their relation to the plantar skin microbial growth Future studies are planned to include the identification of the optimum microclimate with proper internal ventilation during active interventions, which would allow the foot to maintain a balance of microbial colonization and proliferation.

## Supplementary Information

Supplementary Information.

## References

[1] Bouskill L, Havenith G, Kuklane K, Parsons K, Withey W (2002). Relationship between clothing ventilation and thermal insulation. AIHA J..

[2] Quesada P, Kunzler JI, Da Rocha M, Machado EA, Carpes F (2015). Plantar pressure and foot temperature responses to acute barefoot and shod running. Hum. Mov..

[3] Smith CJ, Machado-Moreira CA, Plant G, Hodder S, Havenith G, Taylor NAS (2013). Design data for footwear: Sweating distribution on the human foot. Int. J. Cloth. Sci. Technol..

[4] Irzmańska E, Brochocka A, Majchrzycka K (2012). Textile composite materials with bioactive melt-blown nonwovens for protective footwear. Fibres Textiles East. Eur..

[5] Sarkany I, Taplin D, Blank H (1961). The etiology and treatment of erythrasma. J. Investig. Dermatol..

[6] Ara K, Hama M, Akiba S, Koike K, Okisaka K, Hagura T (2006). Foot odor due to microbial metabolism and its control. Can. J. Microbiol..

[7] Ninomiya J (2000). Effect of temperature, humidity and minor injury to the penetration of dermatophytes into human stratum corneum. Nippon Ishinkin Gakkai Zasshi.

[8] *Lumen: Temperature and Microbial Growth*. https://courses.lumenlearning.com/microbiology/chapter/temperature-and-microbial-growth/. Accessed 01 June 2020.

[9] Chiller K, Selkin BA, Murakawa GJ (2001). Skin microflora and bacterial infections of the skin. J. Investig. Dermatol. Sympos. Proc..

[10] West AM, Schönfisch D, Picard A, Tarrier J, Hodder S, Havenith G (2019). Shoe microclimate: An objective characterisation and subjective evaluation. Appl. Ergon..

[11] Sasagawa Y (2019). Internal environment of footwear is a risk factor for tinea pedis. J. Dermatol..

[12] Irzmańska E (2016). The microclimate in protective fire fighter footwear: Foot temperature and air temperature and relative humidity. Autex Res. J..

[13] Shimazaki Y, Murata M (2015). Effect of gait on formation of thermal environment inside footwear. Appl. Ergon..

[14] Shimazaki Y, Matsutani T, Satsumoto Y (2016). Evaluation of thermal formation and air ventilation inside footwear during gait: The role of gait and fitting. Appl. Ergon..

[15] Cristiani AM, Bertolotti GM, Marenzi E, Ramat S (2014). An instrumented insole for long term monitoring movement, comfort, and ergonomics. IEEE Sens. J..

[16] Morley Jr RE, Richter EJ, Klaesner JW, Maluf KS, Mueller MJ (2001). In-shoe multisensory data acquisition system. IEEE Trans. Biomed. Eng..

[17] Maluf KS, Morley RE, Richter EJ, Klaesner JW, Mueller MJ (2001). Monitoring in-shoe plantar pressures, temperature, and humidity: Reliability and validity of measures from a portable device. Arch. Phys. Med. Rehabil..

[18] Satsumoto, Y., Takeuchi, M., & Havenith, G. The effect of size factor of leather shoes on ventilation rate in shoes. in *The Fourth International Conference on Human Environment System, Sapporo, Japan*. (2011).

[19] Irzmańska E (2014). Case study of the impact of toecap type on the microclimate in protective footwear. Int. J. Ind. Ergon..

[20] Koch W, Kaplan D (1956). A simple method of estimating the ventilation of footwear. Ann. Trop. Med. Parasitol..

[21] Havenith G, Zhang P, Hatcher K, Daanen H (2010). Comparison of two tracer gas dilution methods for the determination of clothing ventilation and of vapour resistance. Ergonomics.

[22] ASTM International (2016). ASTM E96/E96M-16: Standard Test Methods for Water Vapour Transmission of Materials.

[23] Walter IA, Allen RG, Elliott R, Jensen ME, Itenfisu D, Mecham B (2001). ASCE’s standardized reference evapotranspiration equation. Watershed Manag. Oper. Manag..

[24] Monteith J, Unsworth M (2007). Principles of Environmental Physics.

[25] Fierer N, Hamady M, Lauber CL, Knight R (2008). The influence of sex, handedness, and washing on the diversity of hand surface bacteria. Proc. Natl. Acad. Sci. U.S.A..

[26] Qian J, Hospodsky D, Yamamoto N, Nazaroff WW, Peccia J (2012). Size-resolved emission rates of airborne bacteria and fungi in an occupied classroom. Indoor Air.

[27] *Schmidt Laboratory: The Ribosomal RNA Database*. https://rrndb.umms.med.umich.edu/genomes/annotation?query=Bacillus+subtilis&button=Search&select=keyword. Accessed 01 April 2020.

[28] Callahan BJ, McMurdie PJ, Rosen MJ, Han AW, Johnson AJ, Holmes SP (2016). DADA2: High-resolution sample inference from Illumina amplicon data. Nat. Methods.

[29] Quast C, Pruesse E, Yilmaz P, Gerken J, Schweer T, Yarza P, Glöckner FO (2013). The SILVA ribosomal RNA gene database project: Improved data processing and Web-based tools. Nucleic Acids Res..

[30] Yilmaz P, Parfrey LW, Yarza P, Gerken J, Pruesse E, Quast C (2014). The SILVA and “all-species living tree project (LTP)” taxonomic frameworks. Nucleic Acids Res..

[31] Marshall J, Leeming JP, Holland KT (1987). The cutaneous microbiology of normal human feet. J. Appl. Bacteriol..

[32] Stevens D, Cornmell R, Taylor D, Grimshaw SG, Riazanskaia S, Arnold DS (2015). Spatial variations in the microbial community structure and diversity of the human foot is associated with the production of odorous volatiles. FEMS Microbiol. Ecol..

[33] Kandi V, Palange P, Vaish R, Bhatti AB, Kale V, Kandi MR, Bhoomagiri MR (2016). Emerging bacterial infection: Identification and clinical significance of Kocuria species. Cureus.

[34] Leung MHY, Wilkins D, Lee PKH (2015). Insights into the pan-microbiome: Skin microbial communities of Chinese individuals differ from other racial groups. Sci. Rep..

[35] Taylor, N. A. S., Machado-Moreira, C., van den Heuvel, A., Caldwell, J., Taylor, E. A., & Tipton, M. J. The roles of hands and feet in temperature regulation in hot and cold environments. in *Thirteenth International Conference on Environmental Ergonomics*. 405–409. https://ro.uow.edu.au/hbspapers/190 (2009).

[36] Sun P-C, Jao E, Cheng C-K (2005). Assessing foot temperature using infrared thermography. Foot Ankle Int..

[37] Taylor NA, Caldwell JN, Mekjavic IB (2006). The sweating foot: Local differences in sweat secretion during exercise-induced hyperthermia. Aviat. Space Environ. Med..

[38] Bergquist K, Holmér I (1997). A method for dynamic measurement of the resistance to dry heat exchange by footwear. Appl. Ergon..

[39] Kanli, N., Zengin, C. A., & Bitlisli, B. O. The effects of different finishing types on water vapour and air permeability properties of shoe upper leathers. in *Proceedings of the 3rd International Conference on Advanced Materials and Systems, ICAMS 2010, Tamil Nadu, India* (2010).

[40] Irzmańska E, Brochocka A (2014). Influence of the physical and chemical properties of composite insoles on the microclimate in protective footwear. Fibres Textiles East. Eur..

[41] West AM, Oberst F, Tarrier J, Heyde C, Schlarb H, Brüggemann GP, Havenith G (2020). A Thermal Foot Manikin as a Tool for Footwear Evaluation and Development.

[42] Tang YM, Hui KC (2011). Human foot modeling towards footwear design. CAD Comput. Aided Des..

[43] Shen WW, Gu Y, Bull AM (2013). Characteristic of foot surface temperature variety during continual low-intensity exercise. Biotechnology.

[44] Yu AN, Li PL, Yick KL, Ng SP, Yip JN (2018). Investigation of microclimate in sports shoes with the integration of human subjective sensations. Key Eng. Mater..

[45] Tachibana DK (1976). Microbiology of the foot. Annu. Rev. Microbiol..

[46] Findley K, Oh J, Yang J, Conlan S, Deming C, Meyer JA (2013). Topographic diversity of fungal and bacterial communities in human skin. Nature.

[47] Grice EA, Kong HH, Conlan S, Deming CB, Davis J, Young AC (2009). Topographical and temporal diversity of the human skin microbiome. Science.

[48] Bojar R, Holland KT (2002). Review: The human cutaneous microflora and factors controlling colonisation. World J. Microbiol. Biotechnol..

[49] Costello EK, Lauber CL, Hamady M, Fierer N, Gordon JI, Knight R (2009). Bacterial community variation in human body habitats across space and time. Science.

[50] Fukazawa T, Havenith G (2009). Differences in comfort perception in relation to local and whole body skin wettedness. Eur. J. Appl. Physiol..

[51] West AM, Tarrier J, Hodder S, Havenith G (2019). Sweat distribution and perceived wetness across the human foot: The effect of shoes and exercise intensity. Ergonomics.

