# Supplementary material for: Footwear microclimate and its effects on the microbial community of the plantar skin
Source: Sci Rep. 2021 Oct 13;11:20356. doi: 10.1038/s41598-021-99865-x (PMC8514438; doi:10.1038/s41598-021-99865-x)
Supplement: Supplementary file 1 — Supplementary Information. [file 41598_2021_99865_MOESM1_ESM.docx]

**Supplementary Information**


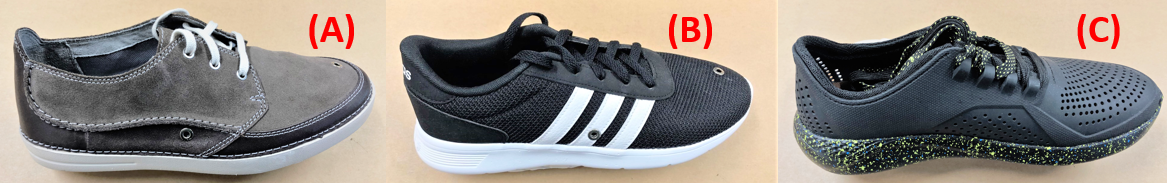


Figure S1: Three footwear types tested during the experiment.

A: casual shoes; B: running shoes; and C: perforated shoes


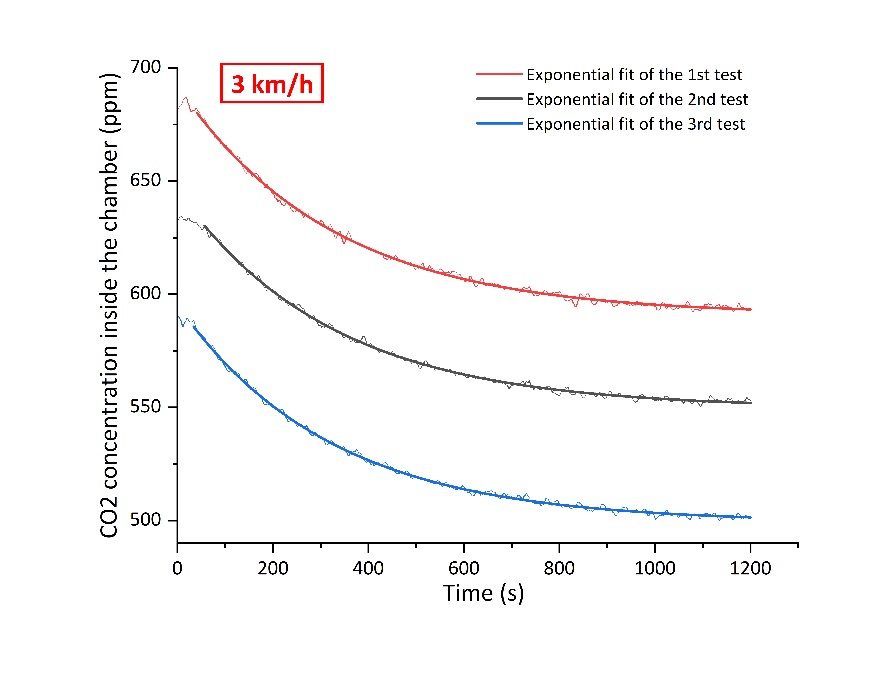

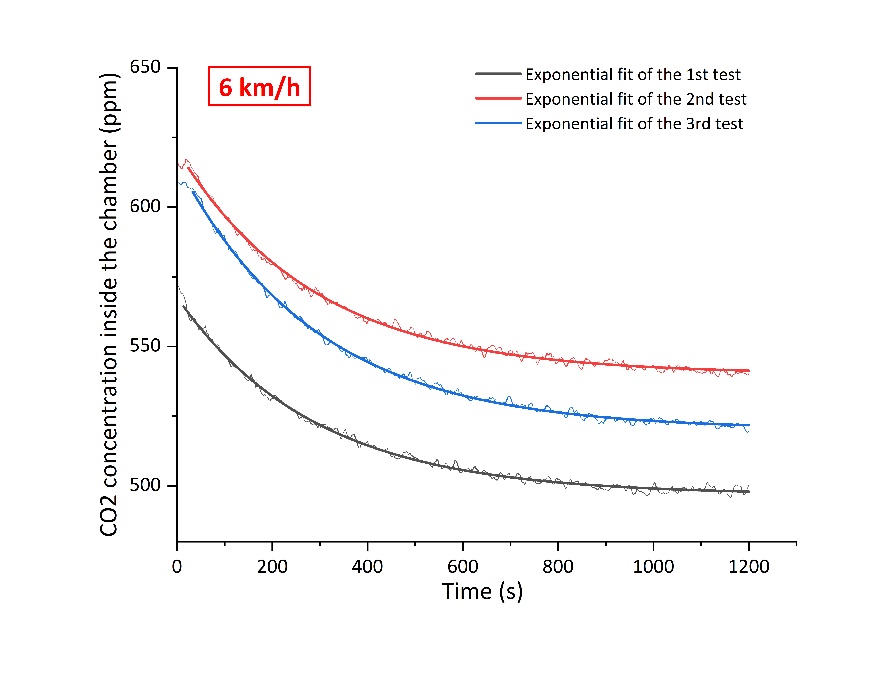


Figure S2: Dilution of CO_2_ concentration inside the chamber as a function of time for the measurement of ventilation rate inside the chamber.

The left figure shows the CO_2_ concentration changes inside the chamber when the set speed of the treadmill was 3 km/h, and the averaged air exchange rate was 11.196 h^-1^. The right figure shows the CO_2_ concentration changes inside the chamber when the set speed of the treadmill was 6 km/h, and the averaged air exchange rate was 12.432 h^-1^. Then the ventilation rate of the chamber was calculated to be 57.47 and 63.82 L/min for the speeds of 3 km/h and 6 km/h respectively when the volume of the chamber was calculated as 308 L.


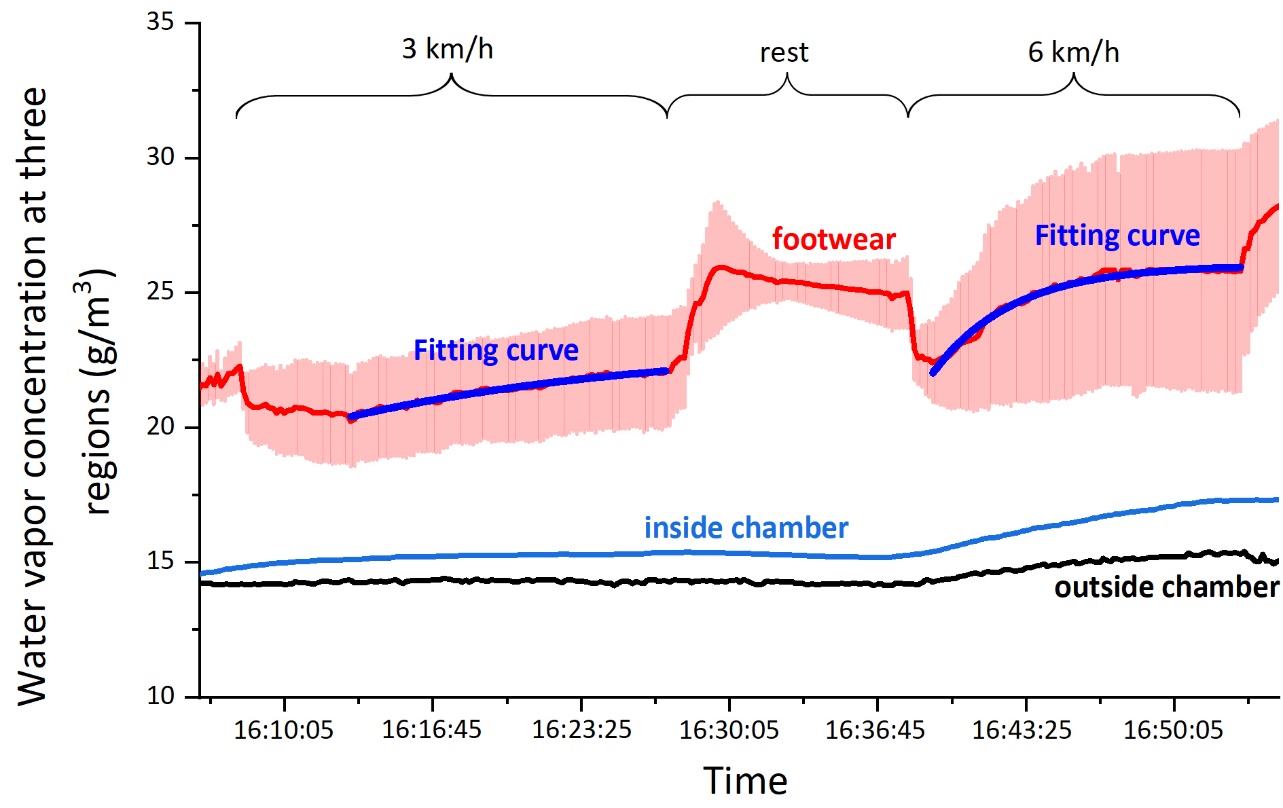


Figure S3: An example of the change in water vapor concentration inside the footwear, inside the chamber, and outside the chamber when exercising on the treadmill at a gait speed of 3 km/h and 6 km/h in sequence with running shoes. The standard deviations of water vapor concentrations inside the footwear at the five measuring locations are presented in the figure.

Fitting curve function derivation process:

The water vapor concentration changes inside the footwear can be expressed as:

$V_{f}\cdot\frac{dM_{f}}{dt}=-Q_{f}\cdot M_{f}+\left( Q_{f}\cdot M_{c}+E_{f}\cdot A_{f} \right)$ (S-1)

$V_{f}\cdot\frac{d\left( M_{f}-M_{c}-E_{f}\cdot A_{f}/Q_{f} \right)}{dt}=-Q_{f}\cdot M_{f}+\left( Q_{f}\cdot M_{c}+E_{f}\cdot A_{f} \right)-V_{f}\cdot\frac{d\left( M_{c}+E_{f}\cdot A_{f}/Q_{f} \right)}{dt}$ (S-2)

Since $\left( Q_{f}\cdot M_{c}+E_{f}\cdot A_{f} \right)\gg V_{f}\cdot\frac{d\left( M_{c}+E_{f}\cdot A_{f}/Q_{f} \right)}{dt}$ according to our experimental results, then the expression (S-2) can be simplified as:

$V_{f}\cdot\frac{d\left( M_{f}-M_{c}-E_{f}\cdot A_{f}/Q_{f} \right)}{dt}=-Q_{f}\cdot\left( M_{f}-M_{c}-E_{f}\cdot A_{f}/Q_{f} \right)$ (S-3)

Therefore, the water vapor concentration inside the footwear can be expressed as: $M_{f}=M_{c}+E_{f}\cdot A_{f}/Q_{f}-\left( -M_{f,0}+M_{c,0}+E_{f,0}\cdot A_{f}/Q_{f} \right)\cdot e^{-\frac{Q_{f}}{V_{f}}\left( t-t_{0} \right)}$ (S-4)

As for the water vapor concentration changes inside and outside the chamber, we can obtain their expressions using a similar derivation process, and the results are shown below:

$M_{c}=\frac{Q_{c}}{Q_{c}+{2Q}_{f}}M_{r}+\frac{2Q_{f}}{Q_{c}+2Q_{f}}M_{f}-\left( -M_{c,0}+\frac{Q_{c}}{Q_{c}+2Q_{f}}M_{r,0}+\frac{{2Q}_{f}}{Q_{c}+{2Q}_{f}}M_{f,0} \right)\cdot e^{-\frac{Q_{c}+{2Q}_{f}}{V_{c}}\left( t-t_{0} \right)}$ (S-5)

$M_{r}=\frac{Q_{r}}{Q_{r}+Q_{c}}M_{o}+\frac{Q_{c}}{Q_{r}+Q_{c}}M_{c}+\frac{\bar{E_{r}}}{Q_{r}+Q_{c}}-\left( -M_{r,0}+\frac{Q_{r}}{Q_{r}+Q_{c}}M_{o,0}+\frac{Q_{c}}{Q_{r}+Q_{c}}M_{c,0}+\frac{\bar{E_{r,0}}}{Q_{r}+Q_{c}} \right)\cdot e^{-\frac{Q_{r}+Q_{c}}{V_{r}}\left( t-t_{0} \right)}$ (S-6)

where $Q_{f}$, $Q_{c}$, and $Q_{r}$ (L/min) are the ventilation rate of the footwear, the chamber, and the room (environmental chamber) respectively. $M_{f}$, $M_{c}$, $M_{r}$, and $M_{o}$ (g/m^3^) are the averaged water vapor concentrations of the air inside the footwear, inside the chamber, inside the room, and outside the room respectively. $V_{f}$, $V_{c}$, and $V_{r}$ (L) are the volume of the footwear gap space, the chamber, and the room respectively. $E_{f}$ (mg/min/cm^2^) is the foot sweat rate and $A_{f}$ (cm^2^) is the area of the right foot skin coved by socks. $\bar{E_{r}}$ (mg/min) represents the total water vapor emissions from all the surfaces inside the room and from the participant’s out-of-the-chamber part (respiration and sweating). Here, we assume that the $M_{o}$, $E_{f}$, $\bar{E_{r}}$ and are constant during the experiment period and the expressions of the three variables of $M_{f}$, $M_{c}$, and $M_{r}$ can therefore be obtained by solving the three equations S-4, S-5, and S-6. And the results can be expressed as the below equation which is named as the ExpDecay3 fitting function in Origin 2018:

$y=y_{0}+A_{1}e^{-\left( x-x_{0} \right)/t_{1}}+A_{2}e^{-\left( x-x_{0} \right)/t_{2}}+A_{3}e^{-\left( x-x_{0} \right)/t_{3}}$ (S-7)

Where $y_{0}$ represents the water vapor concentration under the steady-state condition.


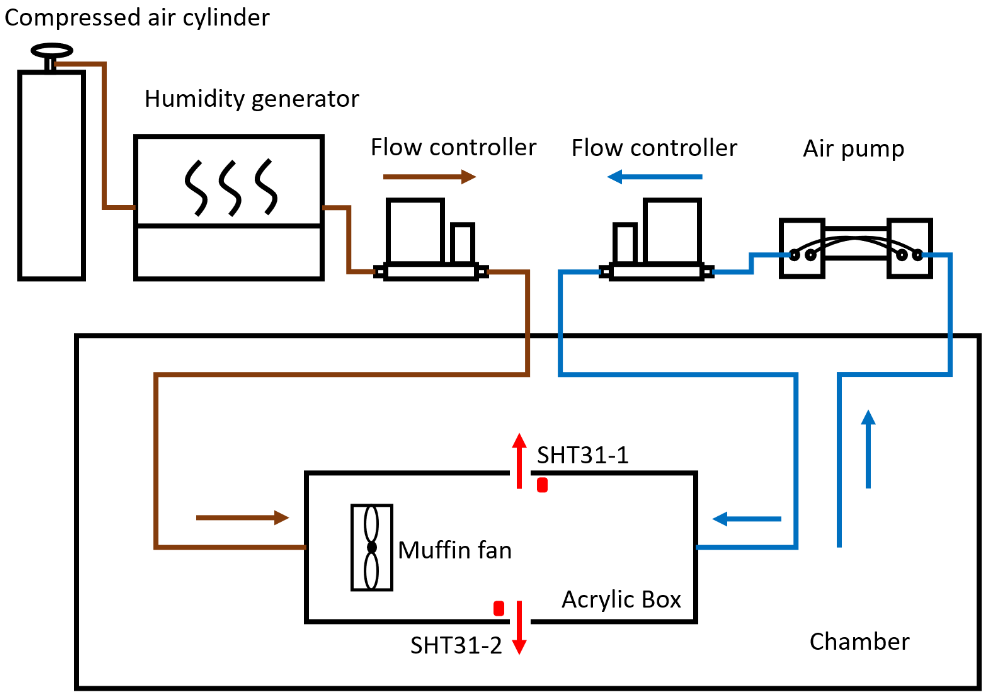


Figure S4: Schematic diagram of the calibration system

The chamber method was calibrated using a sealed acrylic box (100 mm × 100 mm × 280 mm) with two inlets and two outlets, which was put inside the chamber to perform as the “shod feet” as shown above. Moist air from the humidity generator (HumiSys HF, InstruQuest Inc., USA) and return air from the chamber flowed into the box via the two inlets with the known values of flow rates under the control of two mass flow controllers. Mix air was then discharged outside the box to maintain the volumetric balance. The “ventilation rates” of the acrylic box, therefore, could be controlled by adjusting the control valve of the mass flow controller for the return air. Two SHT31 sensors (SHT31-1 and SHT31-2) were placed at the two air outlets to record the box's internal air temperature and humidity. Other setup remained consistent with that of the participant involved experiments.


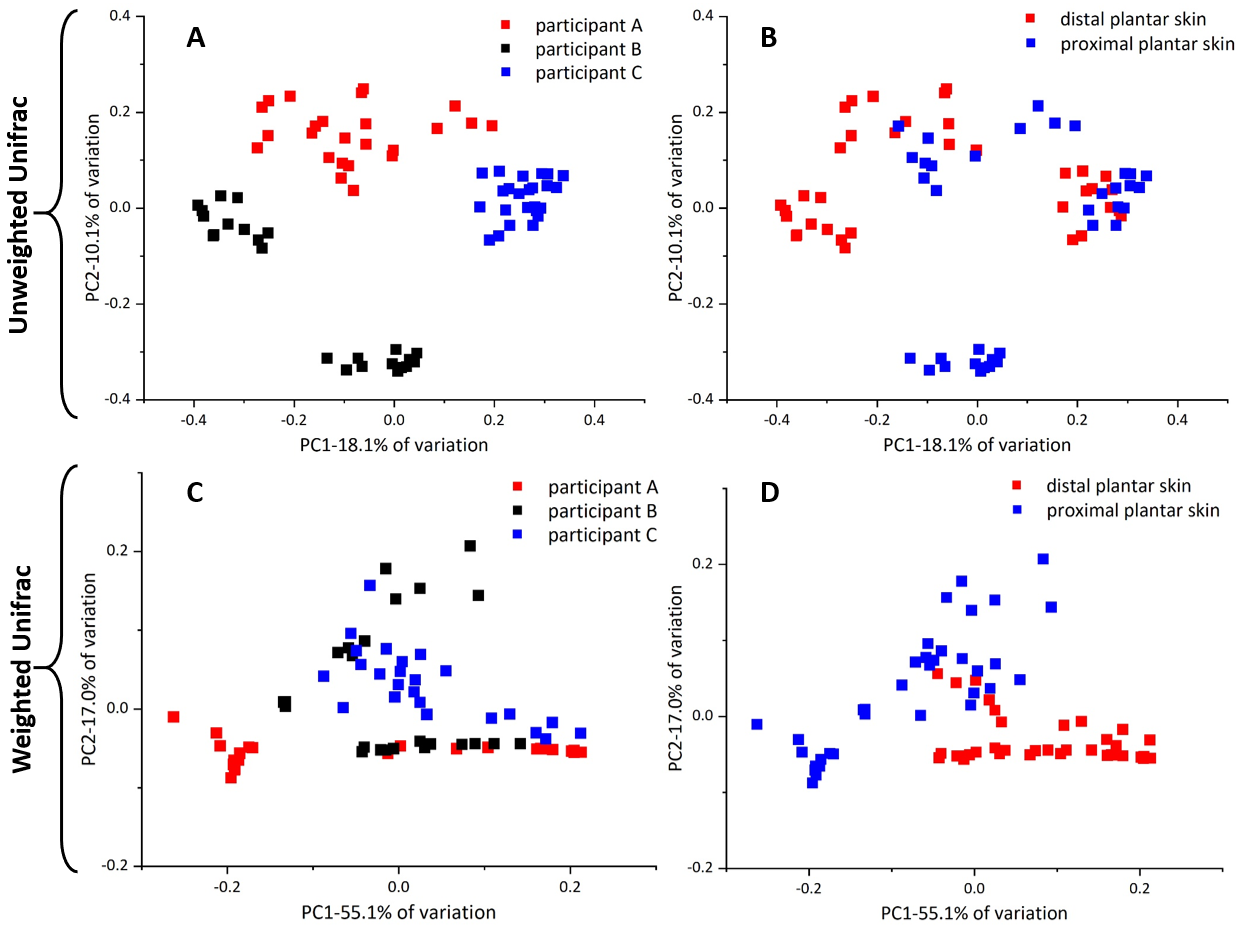


Figure S5: Principal coordinate analysis (PCoA) plots of samples based on pairwise unweighted (A, B) and weighted (C, D) UniFrac distance between samples in community composition, with sample points colored by participants (participant A, participant B, and participant C) and sampling sites (distal and proximal plantar skin).

Table S1: Basic information on the three footwear types.

(Note: no necessary to measure the WVTR of rubber upper since dense holes spreading over the surface)

| **No.** | **Types** | **Brands & Models** | **Upper materials** | **WVTR (g/m^2^.24h)** |
| --- | --- | --- | --- | --- |
| Footwear1 | Casual shoes | Clarks Men’s Gosler Edge Sneaker | Suede | 4177.0981 |
| Footwear2 | Running shoes | Adidas Lite Racer (EH1323) | Textile | 2425.7625 |
| Footwear3 | Perforated shoes | Crocs Men’s LiteRide Hyper Bold Pacer | Rubber with holes | NA |

Table S2: Calibration results for the ventilation rates

| Treadmill speeds | Set vapor generation rate (mg/min) | Set ventilation rates (L/min) | Calculated ventilation rates (L/min) | Slope | Intercept | R^2^ |
| --- | --- | --- | --- | --- | --- | --- |
| 3 km/h | 76.16 | 5 | 3.72 ± 0.97 | 1.0607 | 1.061 | 1 |
|  |  | 10 | 8.42 ± 1.11 |  |  |  |
|  |  | 15 | 13.14 ± 1.30 |  |  |  |
| 6 km/h | 171.35 | 10 | 8.47 ± 0.99 | 0.9186 | 2.1891 | 0.9999 |
|  |  | 15 | 14.02 ± 1.21 |  |  |  |
|  |  | 20 | 19.35 ± 2.03 |  |  |  |

Table S3: Results of the three-way and two-way ANOVA performed in SPSS Statistics V.9.0

(Significant: *p*<0.05)

| **Dependent variables** | | **Independent variables** | | | **Independent variables interaction significance** | |
| --- | --- | --- | --- | --- | --- | --- |
|  |  | (1) | (2) | (3) | (1), (2) & (3) | (2) & (3) |
| Thermal data | Temperature | Individual | Footwear type | Measuring region | 0.105 | <0.001 |
|  | Absolute humidity | Individual | Footwear type | Measuring region | 0.674 | <0.001 |
| Ventilation date | Ventilation rate | Individual | Footwear type | Gait speed | 0.125 | 0.001 |
| Microbial data | Bacterial population | Individual | Footwear type | Skin site | 0.299 | 0.025 |
|  | Bacterial increase ratio | Individual | Footwear type | Skin site | 0.390 | 0.016 |
|  | Bacterial diversity | Individual | Footwear type | Skin site | NA | NA |

Table S4: Bacterial β-diversity significances (*p*-values) between sample variables.

(Significant: *p* <0.05)

|  | **β-diversity significances (*p*-values)** | |
| --- | --- | --- |
| Variables | unweighted Unifrac distance | weighted Unifrac distance |
| Individual | 0.001 | 0.001 |
| Skin site | 0.001 | 0.001 |
| Footwear type | 0.723 | 0.495 |

Table S5: Bacterial β-diversity significances (*p*-values) between the variable “Footwear type” within six subgroups separately, which were grouped based on the variables “individual” and “skin site”.

(Significant: *p*<0.05)

|  | | | **β-diversity significances (*p*-values)** | |
| --- | --- | --- | --- | --- |
| Variables | | | unweighted Unifrac distance | weighted Unifrac distance |
| Footwear type | Participant A | distal skin | 0.704 | 0.006 |
|  |  | proximal skin | 0.794 | 0.355 |
|  | Participant B | distal skin | 0.120 | 0.004 |
|  |  | proximal skin | 0.043 | 0.015 |
|  | Participant C | distal skin | 0.065 | 0.003 |
|  |  | proximal skin | 0.688 | 0.416 |

Table S6: Linear regression results (adjusted R^2^ and significance) between footwear microclimate (ventilation rate, temperature, and absolute humidity) and bacterial increase ratio on distal and proximal plantar skins separately.

(Significant: *p*<0.05)

|  | | **Distal plantar skin** | | **Proximal plantar skin** | |
| --- | --- | --- | --- | --- | --- |
|  |  | Adjusted R^2^ | Significant | Adjusted R^2^ | Significant |
| **Ventilation rate** | 3 km/h | 0.469 | **< 0.001** | 0.008 | 0.282 |
|  | 6 km/h | 0.373 | **0.001** | 0.092 | 0.073 |
| **Temperature** | Toe region (0) | -0.041 | 0.826 | -0.026 | 0.533 |
|  | Medial region (1) | -0.006 | 0.37 | -0.039 | 0.897 |
|  | Lateral region (2) | -0.026 | 0.558 | 0.003 | 0.306 |
|  | Instep region (3) | 0.23 | **0.007** | -0.038 | 0.811 |
|  | Heel region (4) | 0.109 | 0.055 | -0.027 | 0.567 |
|  | 0/1 | -0.008 | 0.38 | -0.022 | 0.515 |
|  | 0/2 | -0.019 | 0.476 | 0.005 | 0.297 |
|  | 0/3 | 0.106 | 0.054 | -0.024 | 0.537 |
|  | 0/4 | 0.012 | 0.262 | -0.038 | 0.825 |
|  | 1/2 | -0.018 | 0.465 | -0.021 | 0.5 |
|  | 1/3 | 0.158 | **0.023** | -0.04 | 0.932 |
|  | 1/4 | 0.059 | 0.117 | -0.036 | 0.761 |
|  | 2/3 | 0.108 | 0.053 | -0.026 | 0.564 |
|  | 2/4 | 0.013 | 0.26 | -0.036 | 0.773 |
|  | 3/4 | 0.318 | **0.001** | -0.024 | 0.543 |
|  | 0/1/2 | -0.015 | 0.436 | -0.012 | 0.417 |
|  | 0/1/3 | 0.084 | 0.078 | -0.031 | 0.64 |
|  | 0/1/4 | 0.02 | 0.227 | -0.038 | 0.846 |
|  | 0/2/3 | 0.061 | 0.114 | -0.012 | 0.417 |
|  | 0/2/4 | 0.002 | 0.317 | -0.028 | 0.597 |
|  | 0/3/4 | 0.152 | **0.025** | -0.039 | 0.903 |
|  | 1/2/3 | 0.07 | 0.098 | -0.032 | 0.654 |
|  | 1/2/4 | 0.015 | 0.248 | -0.038 | 0.813 |
|  | 1/3/4 | 0.205 | **0.01** | -0.034 | 0.703 |
|  | 2/3/4 | 0.155 | **0.024** | -0.04 | 0.925 |
|  | 0/1/2/3 | 0.046 | 0.147 | -0.021 | 0.506 |
|  | 0/1/2/4 | 0.005 | 0.298 | -0.031 | 0.652 |
|  | 0/1/3/4 | 0.112 | **0.049** | -0.039 | 0.908 |
|  | 0/2/3/4 | 0.085 | 0.076 | -0.033 | 0.678 |
|  | 1/2/3/4 | 0.106 | 0.054 | -0.039 | 0.911 |
|  | 0/1/2/3/4 | 0.067 | 0.102 | -0.034 | 0.716 |
| **Absolute humidity** | Toe region (0) | -0.028 | 0.564 | -0.043 | 0.893 |
|  | Medial region (1) | 0.126 | **0.039** | 0.045 | 0.147 |
|  | Lateral region (2) | -0.006 | 0.37 | -0.018 | 0.473 |
|  | Instep region (3) | 0.177 | **0.017** | -0.013 | 0.424 |
|  | Heel region (4) | 0.194 | **0.014** | 0.05 | 0.142 |
|  | 0/1 | 0.08 | 0.083 | -0.035 | 0.724 |
|  | 0/2 | 0.012 | 0.263 | -0.018 | 0.468 |
|  | 0/3 | 0.137 | **0.033** | -0.036 | 0.758 |
|  | 0/4 | 0.103 | 0.057 | -0.027 | 0.575 |
|  | 1/2 | 0.065 | 0.106 | -0.033 | 0.692 |
|  | 1/3 | 0.202 | **0.011** | 0.009 | 0.277 |
|  | 1/4 | 0.188 | **0.014** | 0.057 | 0.121 |
|  | 2/3 | 0.185 | **0.014** | -0.032 | 0.661 |
|  | 2/4 | 0.115 | **0.047** | -0.022 | 0.512 |
|  | 3/4 | 0.238 | **0.006** | 0.016 | 0.242 |
|  | 0/1/2 | 0.057 | 0.121 | -0.04 | 0.989 |
|  | 0/1/3 | 0.159 | **0.023** | -0.024 | 0.532 |
|  | 0/1/4 | 0.134 | **0.034** | -0.008 | 0.384 |
|  | 0/2/3 | 0.136 | **0.033** | -0.039 | 0.905 |
|  | 0/2/4 | 0.086 | 0.075 | -0.038 | 0.82 |
|  | 0/3/4 | 0.181 | **0.016** | -0.018 | 0.465 |
|  | 1/2/3 | 0.19 | **0.013** | -0.017 | 0.459 |
|  | 1/2/4 | 0.136 | **0.033** | -0.003 | 0.345 |
|  | 1/3/4 | 0.23 | **0.007** | 0.028 | 0.199 |
|  | 2/3/4 | 0.219 | **0.008** | -0.009 | 0.391 |
|  | 0/1/2/3 | 0.149 | **0.027** | -0.033 | 0.679 |
|  | 0/1/2/4 | 0.109 | 0.051 | -0.028 | 0.587 |
|  | 0/1/3/4 | 0.187 | **0.014** | -0.006 | 0.369 |
|  | 0/2/3/4 | 0.17 | **0.019** | -0.028 | 0.598 |
|  | 1/2/3/4 | 0.212 | **0.009** | 0.002 | 0.317 |
|  | 0/1/2/3/4 | 0.174 | **0.018** | -0.019 | 0.484 |

Table S7: Results for the tests of normality for various variables

| **In-shoe temperature** | | | | | | | | |
| --- | --- | --- | --- | --- | --- | --- | --- | --- |
|  |  |  | Kolmogorov-Smirnov^a^ | | | Shapiro-Wilk | | |
| Type | Location | Variable | Statistic | df | Sig. | Statistic | df | Sig. |
| Casual shoes | toe | temperature | 0.205 | 7 | 0.200* | 0.951 | 7 | 0.743 |
|  | medial | temperature | 0.283 | 9 | 0.036 | 0.874 | 9 | 0.134 |
|  | lateral | temperature | 0.239 | 9 | 0.147 | 0.864 | 9 | 0.106 |
|  | instep | temperature | 0.148 | 9 | 0.200* | 0.937 | 9 | 0.551 |
|  | heel | temperature | 0.162 | 8 | 0.200* | 0.955 | 8 | 0.758 |
| Running shoes | toe | temperature | 0.150 | 9 | 0.200* | 0.956 | 9 | 0.758 |
|  | medial | temperature | 0.190 | 9 | 0.200* | 0.950 | 9 | 0.691 |
|  | lateral | temperature | 0.252 | 9 | 0.103 | 0.919 | 9 | 0.381 |
|  | instep | temperature | 0.137 | 9 | 0.200* | 0.979 | 9 | 0.958 |
|  | heel | temperature | 0.325 | 9 | 0.107 | 0.826 | 9 | 0.091 |
| Perforated shoes | toe | temperature | 0.187 | 9 | 0.200* | 0.874 | 9 | 0.135 |
|  | medial | temperature | 0.245 | 9 | 0.127 | 0.814 | 9 | 0.089 |
|  | lateral | temperature | 0.202 | 9 | 0.200* | 0.930 | 9 | 0.482 |
|  | instep | temperature | 0.213 | 9 | 0.200* | 0.874 | 9 | 0.137 |
|  | heel | temperature | 0.158 | 9 | 0.200* | 0.935 | 9 | 0.533 |
| *. This is a lower bound of the true significance. | | | | | | | | |
| a. Lilliefors Significance Correction | | | | | | | | |

| **In-shoe humidity** | | | | | | | | |
| --- | --- | --- | --- | --- | --- | --- | --- | --- |
|  |  |  | Kolmogorov-Smirnov^a^ | | | Shapiro-Wilk | | |
| Type | Location | Variable | Statistic | df | Sig. | Statistic | df | Sig. |
| Casual shoes | toe | humidity | 0.245 | 7 | 0.200* | 0.889 | 7 | 0.270 |
|  | medial | humidity | 0.165 | 9 | 0.200* | 0.919 | 9 | 0.382 |
|  | lateral | humidity | 0.319 | 9 | 0.009 | 0.852 | 9 | 0.078 |
|  | instep | humidity | 0.225 | 9 | 0.200* | 0.937 | 9 | 0.552 |
|  | heel | humidity | 0.132 | 8 | 0.200* | 0.973 | 8 | 0.919 |
| Running shoes | toe | humidity | 0.217 | 9 | 0.200* | 0.855 | 9 | 0.085 |
|  | medial | humidity | 0.273 | 9 | 0.052 | 0.826 | 9 | 0.070 |
|  | lateral | humidity | 0.236 | 9 | 0.161 | 0.879 | 9 | 0.153 |
|  | instep | humidity | 0.236 | 9 | 0.160 | 0.917 | 9 | 0.368 |
|  | heel | humidity | 0.199 | 9 | 0.200* | 0.930 | 9 | 0.481 |
| Perforated shoes | toe | humidity | 0.211 | 9 | 0.200* | 0.859 | 9 | 0.093 |
|  | medial | humidity | 0.251 | 9 | 0.109 | 0.918 | 9 | 0.374 |
|  | lateral | humidity | 0.219 | 9 | 0.200* | 0.936 | 9 | 0.545 |
|  | instep | humidity | 0.147 | 9 | 0.200* | 0.942 | 9 | 0.600 |
|  | heel | humidity | 0.149 | 9 | 0.200* | 0.965 | 9 | 0.849 |
| *. This is a lower bound of the true significance. | | | | | | | | |
| a. Lilliefors Significance Correction | | | | | | | | |

| **Ventilation rate** | | | | | | | | |
| --- | --- | --- | --- | --- | --- | --- | --- | --- |
|  |  |  | Kolmogorov-Smirnov^a^ | | | Shapiro-Wilk | | |
| Type | Speed | Variable | Statistic | df | Sig. | Statistic | df | Sig. |
| Casual shoes | 3 km/h | ventilation | 0.158 | 9 | 0.200* | 0.883 | 9 | 0.170 |
|  | 6 km/h | ventilation | 0.159 | 9 | 0.200* | 0.967 | 9 | 0.867 |
| Running shoes | 3 km/h | ventilation | 0.229 | 9 | 0.192 | 0.915 | 9 | 0.353 |
|  | 6 km/h | ventilation | 0.189 | 9 | 0.200* | 0.933 | 9 | 0.509 |
| Perforated shoes | 3 km/h | ventilation | 0.220 | 8 | 0.200* | 0.907 | 8 | 0.336 |
|  | 6 km/h | ventilation | 0.234 | 8 | 0.200* | 0.931 | 8 | 0.524 |
| *. This is a lower bound of the true significance. | | | | | | | | |
| a. Lilliefors Significance Correction | | | | | | | | |

| **Bacterial concentration** | | | | | | | | |
| --- | --- | --- | --- | --- | --- | --- | --- | --- |
|  |  |  | Kolmogorov-Smirnov^a^ | | | Shapiro-Wilk | | |
| Type | Skin site | Variable | Statistic | df | Sig. | Statistic | df | Sig. |
| Casual shoes | distal | concentration | 0.206 | 9 | 0.200* | 0.957 | 9 | 0.763 |
|  | proximal | concentration | 0.226 | 9 | 0.200* | 0.941 | 9 | 0.593 |
| Running shoes | distal | concentration | 0.237 | 9 | 0.156 | 0.872 | 9 | 0.128 |
|  | proximal | concentration | 0.227 | 9 | 0.199 | 0.911 | 9 | 0.320 |
| Perforated shoes | distal | concentration | 0.129 | 9 | 0.200* | 0.955 | 9 | 0.747 |
|  | proximal | concentration | 0.188 | 9 | 0.200* | 0.898 | 9 | 0.240 |
| *. This is a lower bound of the true significance. | | | | | | | | |
| a. Lilliefors Significance Correction | | | | | | | | |

| **Bacterial increase ratio** | | | | | | | | |
| --- | --- | --- | --- | --- | --- | --- | --- | --- |
|  |  |  | Kolmogorov-Smirnov^a^ | | | Shapiro-Wilk | | |
| Type | Skin site | Variable | Statistic | df | Sig. | Statistic | df | Sig. |
| Casual shoes | distal | ratio | 0.184 | 9 | 0.200* | 0.957 | 9 | 0.769 |
|  | proximal | ratio | 0.259 | 9 | 0.082 | 0.869 | 9 | 0.121 |
| Running shoes | distal | ratio | 0.133 | 9 | 0.200* | 0.977 | 9 | 0.946 |
|  | proximal | ratio | 0.157 | 9 | 0.200* | 0.932 | 9 | 0.503 |
| Perforated shoes | distal | ratio | 0.272 | 9 | 0.055 | 0.854 | 9 | 0.082 |
|  | proximal | ratio | 0.311 | 9 | 0.052 | 0.794 | 9 | 0.078 |
| *. This is a lower bound of the true significance. | | | | | | | | |
| a. Lilliefors Significance Correction | | | | | | | | |
